# The Role of TRP Channels in Sepsis and Colitis

**DOI:** 10.3390/ijms25094784

**Published:** 2024-04-27

**Authors:** Kristina A. Dvornikova, Olga N. Platonova, Elena Y. Bystrova

**Affiliations:** I.P. Pavlov Institute of Physiology RAS, 199034 St. Petersburg, Russia; 691442@gmail.com (K.A.D.); olgaplatonova1991@mail.ru (O.N.P.)

**Keywords:** TRP channels, intestine, inflammatory bowel disease, ulcerative colitis, Crohn’s disease, colitis, inflammation, sepsis, endotoxins, LPS, CLP

## Abstract

To date, several members of the transient receptor potential (TRP) channels which provide a wide array of roles have been found in the gastrointestinal tract (GI). The goal of earlier research was to comprehend the intricate signaling cascades that contribute to TRP channel activation as well as how these receptors’ activity affects other systems. Moreover, there is a large volume of published studies describing the role of TRP channels in a number of pathological disorders, including inflammatory bowel disease (IBD) and sepsis. Nevertheless, the generalizability of these results is subject to certain limitations. For instance, the study of IBD relies on various animal models and experimental methods, which are unable to precisely imitate the multifactorial chronic disease. The diverse pathophysiological mechanisms and unique susceptibility of animals may account for the inconsistency of the experimental data collected. The main purpose of this study was to conduct a comprehensive review and analysis of existing studies on transient receptor potential (TRP) channels implicating specific models of colitis and sepsis, with particular emphasis on their involvement in pathological disorders such as IBD and sepsis. Furthermore, the text endeavors to evaluate the generalizability of experimental findings, taking into consideration the limitations posed by animal models and experimental methodologies. Finally, we also provide an updated schematic of the most important and possible molecular signaling pathways associated with TRP channels in IBD and sepsis.

## 1. Introduction

Transient receptor potential (TRP) channels are an extensive group of ion channels that generally reside on the plasma membrane of many cell types. They are involved in sensory perception and both normal and pathological cell functioning. There are approximately 28 TRP channels in different organisms that have been demonstrated to have some structural similarities. These channels are able to operate as non-selective cation channels since their structure includes six transmembrane segments and an identical molecular sensing site. However, TRP sub-families share little structural homology and are unique [1,2]. Due to such particular features, these ion channels possess multiple functions in the body’s regulation and sensory perception.

A considerable amount of literature has been published on the role of TRPs in conditions such as inflammatory bowel disease (IBD), pain, fibrosis and sepsis. The methodological approaches taken in these studies were based on the application of the clinical cases, human cell lines and animal models. It should be highlighted that ideal animal models of inflammatory bowel disease are absent. In the studies where the data have been obtained from cell lines, the impacts of the cellular microenvironment and homeostasis are excluded. These limitations mean that such findings need to be interpreted cautiously. The heterogeneity of patients, with varying risk factors, comorbidities and responses to treatment, represents the major limitation in clinical research. This complexity makes it challenging to extrapolate results and establish a clear causal relationship between TRP activity and inflammatory state.

There has been a paucity of the literature on a comprehensive analysis of TRP channel-related illnesses. The main functions of transient receptor potential channels in the healthy and inflamed intestines, as well as their synergies, are outlined in this review. We also evaluated the experimental data from the literature of recent years, on the basis of which we present a schematic of the main and potential molecular signaling pathways associated with TRP channels in IBD and sepsis in this review.

## 2. Modern Understanding of TRP Channels

TRP channels function as important non-selective cation receptors with a wide range of permeability, taking part in a variety of physiological processes, from the perception of different stimuli to ion homeostasis. They are present in various tissues and organs, and are mainly located in cell and organelle membranes, providing ion entry [1]. 

In humans, six families of TRP channels have been identified, divided into two groups based on differences in sequence and topology: Group 1, which has a large cytosolic domain—TRPC (Canonical), TRPV (Vanilloid), TRPM (Melastatin) and TRPA (Ankyrin), and Group 2, which has a large exoplasmic domain—TRPP (Polycystin) and TRPML (Mucolipin) [2]. Despite many advances in understanding the evolution of TRP, the phylogenetic relationships between TRP channels are still incomplete. TRP channels have several key structural features, in particular, six transmembrane spanning domains (S1–S6) with a pore-forming loop between S5 and S6 and intracellular C- and N-termini [3]. The cytoplasmic end of S6 helix forms the lower gate, which regulates the entry of cations into the channel. The S1–S4 domains can block the pore in response to ligand binding. The channels of Group 2 have high sequence homology in their transmembrane structural domains and differ from Group 1 in that they contain a large extracellular domain between S1/S2 transmembrane helix [4].

To date, research has focused not only on elucidating the complex signaling mechanisms underlying the activation of TRP signaling pathways and the effect of their activity on other systems, but also on investigating the key role of TRP channels in various pathological conditions, especially accompanied by pain and inflammation [1]. Further research in this field may lead to the development of modern pharmacological approaches for pain prevention. 

### 2.1. The Role of TRP Channels in Pain

The TRPV1, TRPV4, TRPA1, and TRPM8 channels are the ones that have been studied most extensively in connection with visceral pain [5]. 

TRPA1 is implicated in various types of pain, such as neuropathic cold pain, inflammatory pain, and hereditary episodic pain syndromes [6]. Activation of TRPA1 channels results in an influx of Ca^2+^ ions, which causes depolarization of the nociceptive nerve terminal, necessary for the generation of a centrally propagating nociceptive signal.

TRPV1 channel, which is widely expressed in bipolar neurons, transmits sensory information from the periphery to the somatosensory cortex via the spinal cord. TRPV1 is involved in the induction of heat-associated pain and also contributes to peripheral sensitization [7]. Upon TRPV1 activation, extracellular Ca^2+^ enters the cells and its intracellular pool is released, thereby increasing the intracellular Ca^2+^ concentration, which subsequently mediates the activity of many cells, bringing effects such as muscle contraction, changes in neuronal activity, neurotransmitter release, cell proliferation and apoptosis, as well as regulation of body temperature and pain [8]. TRPV1 can also oligomerize with other subunits of some TRP family members, including TRPV3 and TRPA1 [9]. As such, in the gastrointestinal tract (GI), TRPV1 is often co-expressed with TRPA1 on capsaicin-sensitive neurons [10].

Another channel, TRPV4, mediates inflammatory and neuropathic pain [11]. Activation of TRPV4 induces transcriptional regulation and release of neuropeptides—substance P (SP) and Calcitonin Gene-Related Peptide (CGRP)—which results in chronic pain. CGRP and SP act as inflammatory regulatory mediators released from sensory nerves, myenteric neurons, and inflammatory cells such as lamina propria macrophages, B cells, and eosinophils [12]. In general, not only TRPV4, but also other TRP channels (specifically, TRPV1, TRPA1, and TRPM8) are capable of triggering CGRP and SP release, thus increasing the local inflammatory response [13]. 

Although TRPV1 and TRPV4 have been the main focus of pain-related research, TRPV2 and TRPV3 are also of scientific interest. TRPV2 is widely expressed in neuronal and non-neuronal cells [14]. However, the role of TRPV2 in sensory neurons is still unknown, and there have been few studies on pain. TRPV3 has been suggested as a potential transducer that could contribute to the development of pain-associated hypersensitivity in inflammation [15]. 

TRPM channels are also associated with pain. In particular, TRPM8, which is expressed on peripheral sensory neurons and is known as a cold temperature sensor, plays a role in herpes infection and in the development of hyperalgesia after chronic trigeminal nerve injury [16]. TRPM8 activation by its agonist menthol aggravates cold hypersensitivity in ION-CCI rats, whereas treatment with TRPM8 inhibitor capsazepine ameliorates cold allodynia/hyperalgesia, thus supporting the idea that TRPM8 is attributed to trigeminal neuropathy. Heat-sensing TRPM2 is involved in inflammatory pain and promotes visceral hypersensitivity by stimulating a number of immune functions [17,18]. The heat-activated ion channel TRPM3 is associated with thermal hyperalgesia and spontaneous pain, but not with cold or mechanical hyperalgesia [19].

The identification of TRP channels in sensory neurons involved in pain generation has led to considering them as potential targets for pharmacological pain relief [20]. This was a turning point in solving the global pain problem. Opioids are known to be beneficial pain relievers, but their effects on the brain can be addictive [21]. The strategy for preventing opioid side effects is to target the starting point of the pain pathway, namely the pain receptors. However, targeting TRP channels for pain relief also has a number of challenges. First, most of them form functional channels as homotetramers, but heteromultimerization is often possible [3]. This condition poses a potential problem for drug discovery, since heteromultimers are difficult to recombine in heterologous expression systems and may have different pharmacological properties. Second, the use of new TRP-based analgesics has side effects (dizziness, hypertension, heat shivering, headache, nausea, diarrhea, chills, decreased body temperature) that may limit drug development. However, in the last decade, several TRP modulators of chemical, biological and natural origin have been clinically tested and used for the treatment of inflammatory, neuropathic and visceral pain, although the therapeutic mechanism of their action is still unclear [22].

It has been reported that co-activation of TRPM8, TRPA1 and TRPV1 by agonists such as capsaicin and menthol may result in an analgesic effect [23]. Although the reduction in nociceptive perception is likely attributed to the co-activation of TRP channels, it is still unclear how the effect is induced and what mechanisms are responsible for this analgesia. It is also unknown whether other pain-related TRP channels act as the sole mediators of nociception.

Studies considering the potential use of TRP antagonists as a therapeutic tool for pain treatment require special attention. TRPV1 antagonists are suggested as a promising approach. However, most TRPV1 antagonists increase body temperature and the threshold for toxic heat (>43 °C), posing a serious problem for drug development. Despite this, the search for new antagonists and ways to avoid side effects remains an important challenge. ABT-102, a TRPV1 antagonist, has undergone phase I clinical trials for the treatment of pain caused by inflammation, tissue damage, and ischemia [24]. ABT-102 was clinically well tolerated with repeated dosing but caused a transient increase in mean body temperature. Recently, AZD1386, another TRPV1 antagonist, has been studied for therapy of neuropathic pain, dental pain, non-erosive reflux disease and osteoarthritis. However, this study ended in phase II due to nonsignificant pain reduction [25]. The results of a phase II clinical trial were obtained for the topical treatment of itching, rosacea and atopic dermatitis with a cream based on TRPV1 antagonist PAC-14028 [26]. The preliminary data indicate that PAC-14028 meets the safety and efficacy criteria. TRPA1 antagonists are also being considered as a therapeutic approach for pain treatment. However, clinical trials of TRPA1 antagonists GRC17536 and GDC-0334, aimed at treating painful diabetic neuropathy, pain and itching, were stopped due to their ineffectiveness in some participants [6,27]. 

The problem with using TRPA1 antagonists is their high lipophilicity and relative insolubility. Additionally, systemic administration of TRP channel antagonists and agonists in clinical practice can lead to various side effects. As a result, ongoing clinical trials may concentrate on the topical application of TRP channel modulators, such as patches, mucosal sprays, and nanodelivery systems. Furthermore, it is essential to enhance the specificity of the developed substances to avoid cross-activation or inhibition of different TRP channels, especially TRPV1 and TRPA1.

### 2.2. The Role of TRP Channels in Inflammation

Special attention is paid to the role of TRP channels in inflammatory processes, in particular in GI inflammation [13]. It has been established that TRP channels are involved in the modulation of human intestinal motility and secretion. Changes in TRP activity are often associated with a wide range of intestinal diseases and pathological conditions, including inflammatory bowel disease (IBD), fibrosis and visceral hyperalgesia [13]. TRPV1 and TRPA1 are the two most commonly studied TRP channels for their effects on intestinal inflammation.

GI inflammation is followed by an increase in TRPV1 and TRPA1 levels, which indicates the importance of these channels in the immune regulation of the inflammatory response [28]. TRPV1 has been shown to play a role in modulating T-cell activation and differentiation. Its protective effects in T-cell-mediated inflammatory diseases have been reported [29]. In addition, TRPV1 is involved in regulating the activation of colonic mucosal dendritic cells and maintaining T-helper 17 (Th17) immune responses to inflammatory stimuli [30]. Furthermore, it has been reported that TRPV1 can be sensitized by various inflammatory mediators. This, in turn, leads to abdominal pain [31]. It is suggested that neuropathic pain during inflammation is associated with TRPV1 activation, as this channel promotes an increase in the intracellular Ca^2+^ concentration and subsequent release of neuropeptides such as SP and CGRP from afferent sensory neurons, as well as key pro-inflammatory cytokines associated with T-helper 2 (Th2), such as tumor necrosis factor alpha (TNF-α), interleukin-4 (IL-4), interleukin-6 (IL-6). In turn, many pro-inflammatory factors, including SP, nerve growth factor (NGF), bradykinin, and prostaglandins, can sensitize TRPV1 [32]. 

TRPA1 is involved in the inflammatory reactions and the development of mechanical and chemical colon hypersensitivity [33]. TRPA1 stimulation induces Ca^2+^-mediated secretion of SP and CGRP. This leads to increased vascular permeability and activation of inflammatory cells, resulting in neurogenic inflammation. Additionally, tissue damage that occurs during inflammatory reactions can lead to the release of various inflammatory agents, such as reactive oxygen intermediates, nitric oxide, prostaglandins, histamine, serotonin, cytokines, neuropeptides, neurotrophins and chemokines. Some of these agents may block or modulate TRPA1 activity, thereby stimulating intracellular signaling cascades that contribute to acute pain [34]. Furthermore, it has been established that TRPA1 located on vascular smooth muscles and macrophages mediates the pro- and anti-inflammatory effects by regulating the release of cytokines such as interferon-γ (IFN-γ), interleukin-2 (IL-2), interleukin-10 (IL-10), and TNF-α [10]. 

There are a few studies on the role of other TRP channels in intestinal inflammation. Specifically, TRPV2 is known to be an important channel for regulating inflammation in the intestinal mucosa [35]. TRPV4 activation in the GI increases the intracellular Ca^2+^ concentration, which ultimately leads to chemokine release and inflammation [11]. TRPV4 plays a pro-inflammatory role and is expressed in intestinal epithelial cells (EC) [36]. The pro-inflammatory effect of TRPV4 activation can be enhanced by stimulation of the main mediator of neurogenic inflammation and pain, protease-activated receptor 2 (PAR2) [37]. 

Furthermore, the role of TRPM2 in inflammation has been investigated as this channel is highly expressed in immune cells [38]. TRPM2 has been shown to contribute to the development and progression of inflammation, exacerbating inflammatory responses and pain [39].

Several TRP channels are crucial in pain nociception and in exacerbating or alleviating inflammatory processes in the GI. However, the effects and consequences of TRP activation in the GI related to pain and inflammation are not yet fully understood and require further research.

## 3. Inflammatory Bowel Disease and Related Problems

Inflammatory bowel disease (IBD) is a group of chronic immune-mediated diseases of the gastrointestinal tract with complex pathophysiology and pathogenesis, the etiology of which remains unclear [40]. IBD encompasses two main disorders: ulcerative colitis (UC) and Crohn’s disease (CD). They differ in their pathophysiology, symptoms, disease course, treatment and potential complications [41,42]. In particular, UC is characterized by an increase in the production of Th2-associated pro-inflammatory cytokines such as interleukin-1 (IL-1), IL-6, interleukin-8 (IL-8) and TNF-α. CD develops due to an excessive response of T-helper 1 (Th1) and Th17 to interleukin-12 (IL-12), interleukin-18 (IL-18) and interleukin-23 (IL-23) produced by antigen-presenting cells (APC) and macrophages [43]. 

Intestinal fibrosis is a major complication of IBD [44]. Transforming growth factor beta (TGF-β) and its receptors are activated in the inflamed intestine, supporting myofibroblast functions in fibrogenesis [45]. 

The treatment options for IBD are constantly changing. Currently, there are numerous effective treatments with fewer side effects and more precise actions. Novel inhibitors targeting cytokines (such as IL-12/23 inhibitors, phosphodiesterase-4 (PDE4) inhibitor), integrins (such as integrin inhibitors), cytokine signaling pathways (such as Janus Kinase Inhibitor (JAK), Mothers against decapentaplegic homolog 7 (SMAD7) blocker) and cell signaling receptors (such as sphingosine-1-phosphate receptor 1 (S1P) modulator) are the preferred approaches for treatment of IBD [46]. 

The study of a recently discovered cytokine, interleukin-27 (IL-27), has revealed both pro-inflammatory and anti-inflammatory properties in various innate and adaptive immunity disorders [47]. However, it is unclear whether IL-27 signaling in the gut has a predominantly protective or harmful effect. Nonetheless, the discovery of IL-27 immunosuppressive properties makes it a promising therapeutic target for treating IBD. 

As such, traditional treatments such as 5-aminosalicylic acid, corticosteroids, immunomodulators, and tumor necrosis factor (TNF)-alpha inhibitors continue to demonstrate therapeutic effectiveness.

The study of IBD in the context of the direct, indirect or mediated role of TRP channels in the development and course of the disease is of particular interest. This section will review the research conducted on the potential impact of TRP channels on disease in individuals with IBD (CD and UC). Additionally, it will examine studies on various animal models of colitis to reflect the differences in results obtained depending on the specific model used.

### 3.1. TRP Channels and IBD in Humans

It has been established that inflammation in IBD can lead to the development of visceral hypersensitivity, and, in some cases, chronic visceral pain [48]. TRP channels are often co-expressed on sensory neurons and are frequently found in biopsies of IBD patients, indicating a potential interrelationship between different TRP [16]. As such, the important role in the development of visceral hypersensitivity and pain in IBD is presumably assigned to TRPA1 and TRPV1 channels [49,50]. Thus, the levels of TRPV1 and TRPA1 in the rectum are significantly increased in IBD, which plays a crucial role in mediating inflammatory pain. This increase affects nerve endings and immune cells, highlighting the importance of TRPV1 and TRPA1 in the immune regulation of the intestinal inflammatory response [28]. At the same time, according to some studies TRPA1 demonstrates anti-inflammatory effects. Specifically, this channel can protect by suppressing the gene expression of pro-inflammatory neuropeptides such as SP, neurokinin A (NKA), and neurokinin B (NKB). Additionally, it inhibits the synthesis of inflammatory cytokines and chemokines produced by macrophages [51]. On the contrary, TRPV1 exhibits mainly pro-inflammatory effects or no effect at all [52]. It is also known that TRPV1 and TRPA1 can form a heterodimer. Apparently, these channels share common physiological characteristics and can mutually influence each other [53]. Due to the great similarity between TRPV1 and TRPA1, it might be expected that these channels have similar functional roles. However, existing results indicate the opposite. The different effects of TRPV1 and TRPA1 may depend on the localization of these channels on specific cell types, the degree of colon inflammation, and the organism type (human or animal model). However, further studies are required to better understand the regulatory role of TRP channels in IBD, including their functional significance and differences, as well as the mechanisms of activation and interaction of TRPV1 and TRPA1 in the inflamed intestine due to the existing contradictions.

#### 3.1.1. TRP Channels, Crohn’s Disease, and Ulcerative Colitis

As previously mentioned, different TRP channels play varying roles in the development of intestinal inflammation. Further, this review will consider the results of studies conducted on intestinal samples from patients with CD and UC.

TRPA1 (but not TRPV1) mRNA was shown to be significantly upregulated in response to inflammation in patients with active and inactive forms of CD and UC compared to healthy controls [28]. TRPA1 activation reduced the expression of several pro-inflammatory neuropeptides, cytokines, and chemokines, leading to a decrease in inflammation. Infiltrating CD4^+^ T cells, which are important mediators of the inflammatory response in IBD, have been found to express TRPA1 and TRPV1 [54]. The largest amount of these cells was found in colon tissue samples with active forms of CD and UC. It has been reported that TRPA1 in CD4^+^ T cells performs a function opposite to that of TRPA1 in visceral sensory neurons [55]. Thus, while neuronal TRPA1 enhances acute inflammation, TRPA1 in CD4^+^ T cells reduces the severity of chronic T-cell-mediated colitis, confirming the protective role of TRPA1 in inflammation. 

The data on TRPV1 are contradictory. On the one hand, TRPV1 promotes colitogenic responses of CD4^+^ T cells and intestinal inflammation [56]. For instance, activation of TRPV1 was observed in the colonic epithelium of patients with active forms of CD and UC [57]. This allows TRPV1 inhibition to be cautiously considered as a new therapeutic strategy to repress pro-inflammatory T-cell responses. However, in another study, TRPV1 expression was significantly reduced in the epithelium of colon samples from UC patients which was accompanied by increased colon inflammation [58]. 

In general, taking into account the crucial role of TRPV1 in chemokine production by epithelial cells and activation of immune cells, it can be assumed that TRPV1 in the colonic mucosa may contribute to the onset and recurrence of IBD. It should also be noted that in UC patients reporting abdominal pain, a positive correlation was found with TRPV1 transcription, but not with mucosal SP concentrations [59]. 

Previous studies have demonstrated that patients with UC in remission who report abdominal pain exhibit elevated TRPV1 levels in the mucosa of the rectosigmoid junction [60]. The results suggest that TRPV1 may be involved in the pathophysiology of ongoing pain and visceral hypersensitivity in this group of patients, indicating a potential therapeutic target. However, there was no difference in TRPV1 immunoreactivity in rectosigmoid junction biopsies when comparing samples from asymptomatic UC patients and healthy volunteers.

High levels of TRPV4 mRNA expression were detected in colon biopsies of patients with CD and UC, resulting in elevated intracellular Ca^2+^ concentrations, release of chemokines, and chronic inflammation [61]. Previous studies have demonstrated that activation of TRPV4 by its agonist 4α-phorbol-12,13-didecanoate (4α-PDD) in human intestinal epithelial cell lines Caco-2 and T84 can affect innate immunity pathways and cause a pro-inflammatory response, exacerbating chronic inflammation [62]. There is also evidence of increased TRPV4 expression in mucosal epithelial cells of patients with UC, which may indicate a possible contribution of this channel to inflammation [58]. According to these findings, it was suggested that TRPV4 is involved in intestinal pathology and could be a significant pharmacological target for treating IBD. 

Few studies have been conducted on human intestinal samples and human epithelial cell lines associated with CD and UC. However, the present results demonstrate that different TRP channels have pro- and anti-inflammatory effects on the development of these two forms of IBD (Table 1). Specifically, TRPA1 exhibited a protective effect by decreasing the production of pro-inflammatory molecules, whereas TRPV1 and TRPV4 channels promoted intestinal inflammation. TRPV1 and TRPV4 antagonists are believed to have therapeutic potential. Further studies are required, including other TRP channels (e.g., TRPM2, TRPM3, TRPM8, TRPV2), which have demonstrated beneficial or negative effects on intestinal inflammation in animal models of colitis, to confirm/refute the severity of these effects in humans.

#### 3.1.2. TRP Channels and Intestinal Fibrosis

As previously noted, intestinal fibrosis results in TGF-β activation in the inflamed intestine to support myofibroblast function in fibrogenesis [45]. Intriguingly, several TGF-β-related pathways intersect with intracellular Ca^2+^ dynamics, which in turn is mediated by various members of the TRP superfamily. TGF-β stimulation was found to increase TRPC6 expression in fibroblasts, and inhibition of calcineurin, a downstream mediator of TRPC6-dependent Ca^2+^ signaling, resulted in decreased myofibroblast differentiation [63]. It is suggested that increased TRPC6 activity is required for TGF-β-mediated myofibroblast differentiation and fibrosis development. Other studies have demonstrated that TRPA1 may have antifibrotic effects [64,65]. It has been reported that knocking down TRPA1 using small interfering RNA (siRNA) leads to enhanced fibrogenic effects when fibrosis is caused by the major profibrotic factor TGF-β. These findings suggest that TRPA1 could be a potential molecular target for antifibrotic therapy in IBD.

### 3.2. TRP Channels and Colitis in Animal Models

Various animal models of colitis have been developed to understand the pathophysiological mechanisms of IBD and to conduct preclinical pharmacological studies [66]. However, it should be noted that no single model of colitis can fully depict the complexity and multifaceted nature of this disease. Despite some limitations, animal models of colitis display similarities and relevance to the histological, pathological, and molecular features of human IBD, making animal studies of this disease an essential approach. The consideration of IBD pathophysiology has been supplemented by studies of the direct, indirect or mediated TRP influence on the development and progression of IBD. This has increased the scale of the problem of a still incompletely studied multifactorial group of chronic inflammatory diseases.

#### 3.2.1. The TNBS-Induced Colitis Model

2,4,6-Trinitrobenzene sulfonic acid (TNBS)-induced colitis is a commonly used animal model that shares properties with human CD and is significant for studying the immunopathogenesis and potential treatments for IBD [67]. 

A rat model of TNBS-induced colitis showed increased expression of TRPM2 in the distal colon [68]. Further, the use of balloon inflation for colonic distension in this colitis model resulted in an increase in visceromotor reflexes and presumably induced visceral hypersensitivity. The increase in visceromotor response caused by balloon pressure was inhibited by oral administration of econazole (TRPM2 inhibitor), which also attenuated visceral hypersensitivity. These findings indicate that neuronal TRPM2 may serve as a mediator and a promising new therapeutic target for the treatment of visceral hypersensitivity associated with intestinal inflammation.

In TNBS-induced experimental colitis in mice, TRPA1 has been found to mediate mechanical hypersensitivity to colonic distension. Additionally, direct stimulation of TRPA1 in nociceptors caused acute pain [69]. Mice lacking functional TRPA1 channels exhibited significantly less inflammation and fibrosis, as well as less severe pain-related behaviors. The reduced inflammation is thought to contribute to less acinar necrosis in TRPA1 null mice. In a rat model of TNBS colitis, the combination of TRPV1 antagonist BCTC and TRPA1 antagonist TCS-5861528 was found to significantly reduce visceral hypersensitivity when compared to the injection of the compounds separately, indicating a synergistic effect [70]. However, the synergistic effect was not observed after the combined intrathecal blockade of TRPA1 and TRPV1. This may indicate a bidirectional pattern of TRP channels functioning.

TRPV1 and TRPV2 expression in the distal colon, dorsal root ganglion (DRG) and nodose ganglion (NG) was significantly increased in a rat model of TNBS-induced colitis [71]. However, no TRPV1 expression was detected in the myenteric plexus. The use of TRPV1 and TRPV2 antagonists alleviated visceral hypersensitivity. These findings suggest that intrinsic/extrinsic TRPV2 and extrinsic TRPV1 neurons contribute to visceral hypersensitivity in an experimental model of TNBS colitis.

The selective blockade of TRPV4 with its antagonist RN1734 in a TNBS mouse model has been shown to alleviate intestinal inflammation and colitis-associated pain [61]. Therefore, inhibiting TRPV4 may have a protective effect against inflammation and could be a potential strategy for treating IBD, either systemically or locally.

#### 3.2.2. The DSS-Induced Colitis Model

The dextran sulfate sodium (DSS)-induced colitis model is known to have limitations and is not a perfect replica of human IBD. However, this model can enhance our understanding of this disease and uncover significant mechanisms and pathways underlying UC pathogenesis in humans [72].

Several studies have shown that the formation of immune infiltrates and colonic ulceration is reduced in TRPM2-deficient mice in a DSS-induced colitis model [73]. In monocytes obtained from TRPM2-deficient mice, Ca^2+^ influx and production of the macrophage chemokine CXCL2, the functional homologue of mouse CXCL8, were impaired. Therefore, TRPM2 may be involved in the progression of colitis through its implication in oxidative stress signaling or other immunomodulatory effects. 

In a recent study on a mouse model of DSS-induced colitis, TRPM3 has been identified in colon-projecting dorsal root ganglion (DRG) neurons [74]. TRPM3 agonist CIM-0216 stimulated DRG neurons and colonic afferents in control animals. Additionally, TRPM3 levels were found to be increased in DRG neurons of mice with colitis. TRPM3 inhibition reduced the excitation of wide-dynamic-range (WDR) neuron afferents in mice with colitis but had no effect in the control group. These results demonstrate that TRPM3 may contribute to the perception of noxious stimuli in colitis, and suggest that the use of TRPM3 antagonists could be a potential approach to reducing colonic hypersensitivity.

Increased number of TRPM8-expressing nerve fibers has been shown to contribute to visceral hyperalgesia in mouse models of DSS- and TNBS-induced colitis [75]. Increased visceral hyperalgesia induced by TRPM8 agonist WS-12 may correlate with upregulation of TRPM8-expressing nerve fibers in the colonic mucosa of mice. It was found that TRPM8 activation by its agonist icilin results in anti-inflammatory effects in a mouse model of DSS-induced colitis [76]. Icilin-dependent activation of TRPM8 blocked CGRP release after TRPV1 activation by capsaicin. Accordingly, the anti-inflammatory effect of icilin is believed to be partly due to its ability to prevent the release of inflammatory neuropeptides. Since TRPV1 expression is increased in IBD and its activation leads to SP and CGRP release triggering neurogenic inflammation, icilin-mediated blockade of TRPV1 function may be beneficial for IBD therapy in humans. The study on a DSS-induced colitis model has demonstrated that wild-type mice treated with repeated menthol (TRPM8 agonist) enemas were protected from DSS colitis. In contrast, *TRPM8*-deficient mice showed increased susceptibility to colitis [77]. Furthermore, the adoptive transfer of *TRPM8*-deficient macrophages was found to exacerbate colitis. Additionally, TRPM8 in macrophages plays a crucial role in determining their pro- or anti-inflammatory effect by regulating the production of tumor necrosis factor α (TNF-α) and IL-10. These findings reveal a novel immunomodulatory effect of TRPM8, suggesting a potential role for this channel in innate immunity.

It was demonstrated that the expression of pro-inflammatory cytokines interleukin-1B (IL-1B), IL-6, interleukin-7 (IL-7), IL-12, IL-23, TNF-α and IFN-γ was higher in mouse colon tissue with mutation *Trpv1G564S^+/+^* than in the control group. This suggests an increased TRPV1 function and susceptibility of mice to DSS-induced colitis [30]. The proportion of DRG neurons expressing TRPV1 in a DSS colitis model and their relative mRNA levels were shown to increase with the subsequent increased release of CGRP and SP [78]. Furthermore, CGRP and SP release induced by TRPV1 activation was greater in the distal colon compared to the proximal colon, possibly indicating the severity of inflammation. Another study demonstrated chronic effects of SP on colonic primary afferents, which could sensitize TRPV1 and lead to persistent abdominal pain following acute inflammation in a mouse model of DSS colitis [31]. At the same time, TRPV1 deletion prevents the development of post-inflammatory visceral hypersensitivity and pain-related behavior. These results suggest regulation of TRPV1 by SP, which may be involved in long-term post-inflammatory visceral pain.

The role of TRPV2 in colitis development has been studied using a mouse model of DSS colitis [35]. Less severe colitis was observed in *TRPV2*^−/−^ mice compared to wild-type animals. This reduction in intestinal inflammation may be due to a decrease in macrophage recruitment, as TRPV2 deficiency has previously been shown to impair the motility of macrophages, T-helper cells and NK cells [79]. However, the exact mechanisms of TRPV2 activity in the intestinal mucosa during inflammation remains unclear.

DSS colitis was significantly attenuated in *TRPV4*-deficient mice compared to wild-type animals. At the same time, intrarectal administration of TRPV4 agonist GSK1016790A exacerbated the severity of DSS colitis [36]. This result demonstrates the pro-inflammatory role of TRPV4 in the intestine and the potential therapeutic value of TRPV4 antagonists in the treatment of chronic intestinal inflammation.

The protective role of TRPA1 in DSS colitis was demonstrated based on histological evaluation and confirmed by TRPA1-mediated suppression of pro-inflammatory neuropeptides and cytokines [28]. Another study has suggested that SP release from sensory neurons expressing TRPV1 or TRPA1 may contribute to the progression of DSS-induced colitis, wherein CGRP provides protection against colon inflammation independently of TRPV1 and TRPA1 expression on sensory neurons [80]. As such, in *TRPV1*- and *TRPA1*-deficient mice, induced DSS colitis was significantly attenuated compared to wild-type animals. These results demonstrate a pro-inflammatory role for TRPA1, which contradicts the findings of previously reviewed studies. Such differences can presumably be explained, inter alia, by the phase of colitis, as well as by different experimental conditions. It was demonstrated that in a mouse model of DSS-induced colitis pretreated with TRPA1 antagonist HC-030031, TRPA1 agonist allyl isothiocyanate (AITC) induced visceral hypersensitivity mediated by colonic chemical stimulation [81]. It is important to note that sensitivity to AITC is not the result of hypersensitivity to mechanical stimuli associated with the intracolonic administration procedure.

#### 3.2.3. Other Models of Colitis

Studies on other animal models of colitis or colitis-like inflammation are also conducted, since there is a need for comprehensive research on the role of TRP channels in colon inflammation and identification of new effects, including negative ones.

Capsaicin-induced sensory denervation of TRPV1 was found to result in excessive neutrophil accumulation in a neonatal mouse model of oxazolone-induced colitis [82]. However, no significant changes in CGRP and SP expression were detected, indicating that mechanisms underlying the progression of colitis presumably regulate pathways other than sensory neuropeptide-induced neurogenic inflammation. Therefore, further research is needed to understand the effects of CGRP and SP on intestinal inflammation.

Wild-type mice with oil of mustard (OM)-induced colitis have been reported to show an increase in levels of neuronal TRPA1, while TRPV1 activation in the colon was not significant [83]. During the 72 h period of colitis induction, the mRNA levels of various neuropeptides and mediators increased. Specifically, cytokines and chemokines such as IL-1B, IL-6, GM-CSF and MCP-1, which are associated with pain and inflammation, were found to be increased. These results suggest the presence of a neurogenic component in OM colitis, combined with an inflammatory component associated with myeloid cells, independent of T and B cells.

A dinitrobenzene sulfonic acid (DNBS)-induced colitis model revealed severe inflammation in *Trpv1*^−/−^-deficient mice, indicating that TRPV1 is required to modulate sensory pathways that regulate the response following the onset of colonic inflammation [84].

Three channels TRPA1, TRPM2 and TRPV1 have been shown to influence apoptosis in mice with colitis-associated colon cancer (azoxymethane (AOM)/DSS model) [85]. Thus, activation of these channels caused increased apoptotic and oxidative effects in colon cancer cells, wherein their inhibition with *Sambucus ebulus* L. reduced the pro-inflammatory effect.

Based on the available evidence, it is apparent that the results obtained from various animal models of colitis are contradictory. These discrepancies arise due to the diverse effects of the colitis-inducing substance on the severity of colitis and the consequences of intestinal inflammation. Furthermore, the contradictions can be attributed to differences in experimental conditions, variations in the concentration of the administered colitis-inducing substances, and small sample sizes. The studies were mainly conducted on the DSS-induced colitis model. In this model, TRPM2, TRPV1, TRPV2, and TRPV4 channels have demonstrated pro-inflammatory effects, while TRPM3 has increased hypersensitivity. TRPM8 and TRPA1 have shown both pro- and anti-inflammatory effects. The results obtained for the TNBS-induced colitis model are partially confirmed by the DSS colitis model, and new findings are also presented. Thus, TRPM2, TRPV1, TRPV2, and TRPV4 promote visceral hypersensitivity and intestinal inflammation, as well as TRPA1 which demonstrates pro-inflammatory effects. The molecular mechanisms underlying the synergy of different TRP channels require further study. Additional research is also needed for TRP channel antagonists, which have demonstrated therapeutic potential. Conflicting results have been shown in rare colitis models induced by oxazolone, DNBS, AOM and OM, particularly regarding the protective effect of TRPV1 channel and the absence of CGRP and SP effect on intestinal inflammation. These findings need confirmation through further investigation, either using the same colitis model with more samples, other colitis models or human samples.

**Figure 1:**
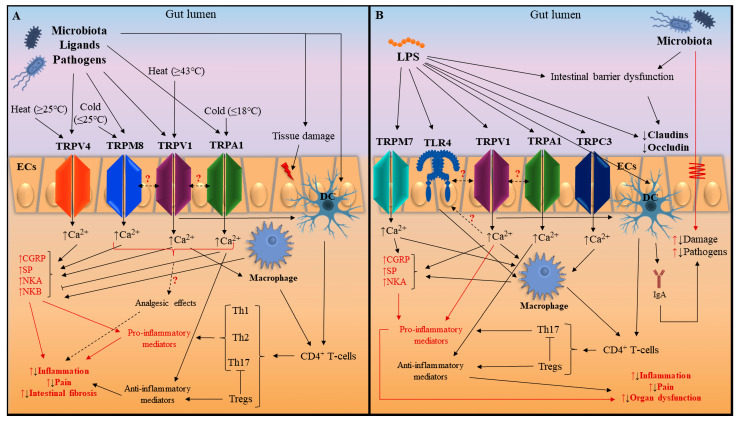
Major and possible molecular pathways associated with TRP channels in IBD and sepsis. The dotted lines indicate the suggested paths. Blunt arrows (┴) indicate inhibition. The red arrows («↑») indicate upregulation and the black arrows («↓») show downregulation associated with TRP molecular pathways. Symbol «?» reflects putative interactions. The red font lettering represents elements of the molecular pathways involved in the pro-inflammatory response. (**A**) Schematic representation of the molecular pathways involved in IBD; (**B**) Schematic representation of the molecular pathways involved in sepsis. ECs, epithelial cells; DC, dendritic cell; Th1, Th2, Th17, T-helper cells; TLR4, Toll-like receptor 4; Tregs, regulatory T cells; IgA, immunoglobulin A; CGRP, Calcitonin Gene-Related Peptide; SP, substance P; NKA, neurokinin A; NKB, neurokinin B (original scheme).

When comparing the findings in different animal models of colitis with the limited results in human intestinal samples and human epithelial cell lines in CD and UC, similar outcomes are reported. Thus, TRPA1 may exhibit a protective effect, while TRPV1 and TRPV4 channels enhance intestinal inflammation in both humans and animals. However, according to existing data on other TRP channels and the observed effects in animal colitis models, there is a need to study positive/negative events associated with TRPM2, TRPM3, TRPM8, and TRPV2 channels in humans (Figure 1, Table 2).

## 4. Sepsis: A Contemporary Menace

Sepsis is a complex pathological process, which is based on the body’s response in the form of systemic inflammation to infections of various origin resulting in acute organ dysfunction [86]. Sepsis is associated with systemic inflammatory response syndrome (SIRS) to endotoxins, and septic shock is its most severe form. Clinical manifestations of sepsis start with inflammation and progress to circulatory dysfunction. Despite advances in understanding the pathogenesis of sepsis and the availability of supportive treatments, sepsis mortality rates remain high globally. The reason is not only the antibiotic resistance of pathogens and immunosuppression caused by sepsis, but also the poorly understood sepsis pathophysiology and low awareness of the consequences of systemic inflammation.

The main infectious agents associated with sepsis are typically members of the ESKAPE pathogen group. This group is the primary cause of nosocomial infections worldwide and includes *Enterococcus faecium*, *Staphylococcus aureus*, *Klebsiella pneumonia*, *Acinetobacter baumannii*, *Pseudomonas aeruginosa*, *Enterobacter* spp. [87]. Studying the molecular mechanisms of antimicrobial resistance in pathogens can facilitate the development of new clinical methods to neutralize key molecules involved in the onset and progression of sepsis [88]. In addition to studying the features of sepsis-inducing pathogens, research is focused on understanding the molecular and signaling pathways that underlie the human immune system response to an infectious agent [89]. In sepsis, the immune response triggered by the invading pathogen results in the development of a pathological syndrome. This syndrome is characterized by persistent excessive inflammation and immune suppression. In the early stages of sepsis, the innate and adaptive immune systems release a range of inflammatory cytokines to eliminate pathogens. Cells of the innate immune system express pattern recognition receptors (PRRs) to identify pathogen-associated molecular patterns (PAMPs) and damage-associated molecular patterns (DAMPs) of different pathogens and molecular structures [90]. Of these, Toll-like receptors (TLRs), NOD-like receptors (NLRs), and RIG-I-like receptors (RLRs) play an essential role in inflammatory responses by recognizing extracellular or endosomal molecular patterns associated with pathogens, resulting in the release of pro- and anti-inflammatory molecules. Anti-inflammatory cytokines include IL-4, IL-10 and interleukin-37 (IL-37). IL-4 induces differentiation of CD4^+^ T cells into Th2 and promotes autocrine mast cell signaling. It also stimulates the release of other anti-inflammatory cytokines while inhibiting the release of pro-inflammatory IL-2 and IFN-γ through activated Th1 [91]. IL-10 exacerbates immunosuppression by reducing the release of pro-inflammatory cytokines, such as TNF-α, inhibiting CD4^+^ T-cell proliferation, and promoting the differentiation of CD4^+^ T cells into regulatory T cells (Tregs). IL-37 is associated with the severity of sepsis-induced immunosuppression by inhibiting the release of pro-inflammatory cytokines from monocytes and neutrophils. Sepsis-induced immunosuppression is mediated by disorders of both innate and adaptive immunity [92]. Further, monocytes exhibit a decreased ability to produce pro-inflammatory cytokines in response to endotoxin exposure, and CD4^+^ T-cell proliferation is also decreased. Prolonged sepsis results in immune cell dysfunction, including impaired release of anti-inflammatory cytokines, T-cell depletion, and overproduction of immunomodulatory cells such as Tregs and myeloid-derived suppressor cells (MDSC). Innate immune dysfunction and suppression of adaptive immunity are responsible for multiple organ damage and septic death [93]. Therefore, it is equally important to maintain a balance between inflammatory and anti-inflammatory responses, as well as normal innate and adaptive immune functions.

### 4.1. The Gut Microenvironment in Sepsis

It is believed that GI plays a crucial role in the pathophysiology of sepsis by simultaneously promoting and maintaining multiorgan dysfunction [94]. The gut microenvironment comprises three interacting elements: the epithelium, the local immune system, and the intestinal microbiome. These elements absorb nutrients and maintain a barrier to prevent the penetration of intraluminal microbiota or their products [95]. The microenvironment is protected by a balance of three elements. However, during sepsis, an imbalance occurs, resulting in pathological changes that cause localized damage and an inflammatory and anti-inflammatory response. This collectively favors the progression of the disease and exacerbates organ dysfunction. Therefore, the gut may play a role not only in mediating intra-abdominal sepsis but also in the spread of extra-abdominal sepsis. As such, it is known that abdominal sepsis is a severe complication associated with SIRS that can lead to destructive processes in the organs of the abdominal cavity and retroperitoneal space [96].

Sepsis can cause dysfunction of the intestinal barrier, resulting in impaired permeability [97]. The intestinal barrier maintains selective permeability through the apical tight junction, which contains multiple isoforms of claudin, occludin, and ZO-1 [97]. However, sepsis causes disturbances in the intestinal tight junctions, leading to changes in the cellular localization of claudins 1, 3, 4, 5, and 8, as well as the activation of claudin 2, resulting in increased permeability [97]. It has also been shown that myosin light chain kinase (MLCK), which leads to the contraction of the actin–myosin ring, is activated in sepsis and increases paracellular permeability. This is accompanied by elevated levels of IL-6, TNF, and IL-1B [98]. 

The interaction between the microbiome and the immune system is complex. Subsets of T-helper cells, especially Th17 and Tregs, are involved in protecting the intestine from pathogenic influences and immune-mediated damage in dysregulated inflammation [99]. Th17 prevent intestinal infections by attracting neutrophils to the intestinal barrier and promoting the elimination of pathogens. On the other hand, Tregs suppress Th17 responses, preventing excessive inflammation that can cause tissue damage. Dendritic cells induce differentiation of CD4^+^ T cells and stimulate the production of cytokines IFN-γ and IL-10, which control inflammation under conditions of constant exposure to intestinal antigens [100]. Recognition of intestinal pathogens by dendritic cells results in the production of pathogen-specific Immunoglobulin A (IgA) in Peyer’s patches, which specifically inhibits the overgrowth of bacterial pathogens [101]. In sepsis these mechanisms are impaired and the later stages of sepsis are associated with cell apoptosis, hyperinflammation, and pathogenic bacteria expansion [102]. 

At the moment, the exact mechanisms responsible for changes in the gut microenvironment caused by sepsis remain unclear. Further research is needed, particularly to enhance our comprehension of the molecular basis for the development of abdominal sepsis.

### 4.2. TRP Channels as Sensors of Bacterial Endotoxins

The results of studies demonstrate that pathogen detection depends not only on the signaling mechanisms of PRRs expressed in immune cells. Additionally, the data reflect another role of TRP channels as sensors of bacterial endotoxins [103]. Sepsis is known to cause somatic or visceral pain, in addition to severe inflammation. These symptoms are associated, among other things, with TRP channels. Their activation increases the concentration of intracellular Ca^2+^, which mediates the release of mediators, ultimately leading to pain. Intracellular Ca^2+^ acts as a secondary messenger and is involved in the TLR4-dependent immune response to bacterial endotoxin [104]. However, the mechanisms underlying the inflammatory response and the increase in intracellular Ca^2+^ in response to pathogens are poorly understood. The intersection of TLR4 and TRP signaling pathways, as well as the TRP-mediated effect on sepsis development and the immune response identified to date, represent a promising area of research. This will bring us closer to understanding the pathophysiology of sepsis and open new therapeutic approaches for the treatment of systemic inflammation [105]. 

It has been established that TRP channels are capable of recognizing lipopolysaccharide (LPS) from *Escherichia coli* before the initiation of TLR4 signaling during inflammation. This can lead to acute neurogenic inflammation and pain induced by the bacterial endotoxin [106]. LPS binding to TLR4 activates a complex of intracellular signaling pathways involving mitogen-activated protein kinase (MAPK) and nuclear factor kappa-light-chain-enhancer of activated B cells (NF-kB), activating protein-1 (AP-1) and Interferon regulatory factor 3 (IRF-3) transcription factors that play a crucial role in the inflammatory response. As such, pain and acute vascular responses, including neurogenic inflammation with CGRP release, depend on the activation of TRP channels in nociceptive sensory neurons and develop independently of TLR4 activation. It is noteworthy that TRP channels can be a functional target of LPS, which suggests that they may play an important role in linking the stimulation of sensory afferent fibers and immune responses during sepsis. However, the precise mechanism of how LPS activates TRP in the gut and its resulting effects are yet to be fully understood. Additionally, the interaction between intracellular signaling pathways triggered by TLR4 activation and TRP activation remains an unresolved issue.

Further, we will review studies that examine the potential influence of TRP channels on the development of sepsis in humans, as well as studies in various animal models of sepsis. This will reflect the differences in results obtained depending on the chosen model and the object of study.

### 4.3. TRP Channels and Sepsis in Humans

Currently, there is limited research on the impact of TRP channels on sepsis in humans. Studies in this area primarily utilize human cell lines. 

Previous research has investigated the role of TRPM2 in LPS-induced cytokine production by human THP-1 monocytes [38]. LPS application resulted in an increase in intracellular Ca^2+^ concentrations and TRPM2 expression in THP-1 cells. TRPM2 regulated LPS-induced production of pro-inflammatory cytokines IL-6, IL-8, IL-10, and TNF-α, allowing Ca^2+^ to pass through the plasma membrane. Considering the pro-inflammatory effects of TRPM2 in IBD, this channel could also be a potential target for sepsis therapy. 

It was shown that LPS impairs plasma membrane integrity and activates the TRPV1, TRPM3 and TRPM8 channels, while TRPV2 remains unaffected on human embryonic kidney HEK293T cells [103]. Furthermore, the disruption of the plasma membrane induced by LPS led to a non-selective passive entry of Ca^2+^ from the extracellular solution, resulting in a non-specific increase in intracellular Ca^2+^ in HEK293T cells. It is worth noting that LPS can elicit significantly stronger responses in HEK293T cells transfected with human TRPV1 than in untransfected cells. The results suggest that TRP channels may function as LPS sensors, which can compromise membrane stability. Additionally, TRPV1 may be directly involved in pain and inflammation associated with bacterial infection.

It has been shown that overexpression of TRPC3 channel in HEK293 cells expressing human TLR4/MD2/CD14 sequences (HEK-TLR4 cells) resulted in a significant increase in the expression levels of pro-inflammatory genes PTGS2, TNFA and IL-12B following LPS treatment compared to the control [107]. HEK-TLR4 cells transfected with G652A-TRPC3 mutant exhibited decreased levels of PTGS2, TNFA, IL-1B, and IL-12B compared to cells transfected with the wild-type TRPC3 construct after LPS treatment. These results suggest that TRPC3 channel plays a role in LPS-activated inflammatory reactions and the immune response to bacterial endotoxins.

It is worth noting that the few studies conducted on human cell lines have shown the significant role of TRP channels, specifically TRPM2, TRPM3, TRPM8, TRPV1, and TRPC3, in identifying bacterial infectious agents and the immune response (Table 3). Further research will enhance our comprehension of the molecular mechanisms underlying TRP channel function as bacterial endotoxin sensors, and will bring us closer to developing improved sepsis therapy using TRP channel antagonists.

### 4.4. TRP Channels and Sepsis in Animal Models

Animal models are a useful alternative to study the cellular and molecular mechanisms underlying sepsis. Abdominal sepsis is a severe complication associated with SIRS, leading to destructive processes in abdominal and retroperitoneal organs. The animal models suggest inducing abdominal sepsis to imitate this process in humans [96]. Currently, the most common experimental models of abdominal sepsis include injection of a toxic agent, such as intraperitoneal or intravenous injection of LPS, which leads to systemic activation of the innate immune system; injection of live pathogens, known as the bacterial inoculum model; disruption of barrier tissue integrity, specifically the colon ascendens stent peritonitis (CASP) model, which reproduces the clinical picture of polymicrobial acute diffuse peritonitis, and cecal ligation and puncture (CLP), which reflects the clinical course of intraperitoneal abscess and polymicrobial peritonitis [108]. However, it should be noted that animal models of sepsis do not encompass all clinical aspects of this complex pathology [109]. 

This section reviews studies investigating the role of TRP channels in the development of sepsis in animal models. However, there are very few studies examining the possible contribution of TRP channels to the onset and progression of abdominal sepsis. Therefore, there is a need for experimental work in this direction. The findings will confirm or refute existing research and provide new insights into the role of TRP channels in sepsis.

#### 4.4.1. A Model of LPS-Induced Sepsis

In order to model sepsis, one of the simplest methods is to administer LPS intravenously or intraperitoneally. LPS induces systemic inflammation, resulting in the production of pro-inflammatory cytokines TNF-α and IL-1, and multiorgan failure with high mortality [110]. However, the use of the LPS-induced sepsis model has limitations. Therefore, caution should be exercised when extrapolating results from this model to human sepsis. Specifically, the model sepsis induced by endotoxin of Gram-negative bacteria is characterized by rapidly increasing cytokine levels. In contrast, human pathology is associated with prolonged cytokine elevation.

The mechanism of TRP channel activation by LPS remains unclear. However, LPS has been shown to naturally activate TRPA1 [111]. The type of LPS affecting the channel determines the consequences of TRPA1 activation. For instance, *E. coli* LPS causes more significant structural changes in the plasma membrane than LPS of *Salmonella minnesota*. It was found that TRPA1 can recognize *E. coli* LPS independently of TLR4, which activates sensory neurons in mice isolated from the nodose and trigeminal ganglia, leading to acute neurogenic inflammation and pain [106]. Based on these results, it appears that TRPA1 plays a role in the initial response to pathogens by detecting potentially harmful compounds and triggering the production of pro-inflammatory cytokines.

TRPV1 can also be activated by LPS, but less efficiently than TRPA1 [103]. It has been demonstrated that LPS significantly enhances the sensitivity of TRPV1 to capsaicin. This effect can be blocked by a selective TLR4 antagonist [112]. This indicates the ability of LPS to directly activate trigeminal neurons in mice and sensitize TRPV1 through a TLR4-mediated mechanism. 

Thus, it seems that TRPA1, TRPV1, and TLR4 act as synergistic sensors to detect the presence of pathogens. However, the exact mechanism of LPS-induced activation of TRPV1 and TRPA1 in the gut and its consequences still remains to be clarified. 

Although the role of TRPV1 in inflammation, including sepsis, is mainly pro-inflammatory, there is evidence suggesting its anti-inflammatory effects in animal models of sepsis [113]. 

It was shown that TRPV1 activation was associated with visceral pain in LPS-induced peritonitis in mice [114]. Furthermore, in the late phase of peritonitis, TRPV1 was found to have an anti-inflammatory effect on the spleen, resulting in reduced levels of pro-inflammatory cytokines TNF-α, IL-6, IL-10 and IFN-γ. This effect is believed to be mediated by the activation of the sympathetic nervous system and/or noradrenergic neurons induced by LPS-dependent TRPV1 activation. The neuropeptides responsible for the protective effect of TRPV1 in sepsis and the mechanisms of their activation are yet to be determined. However, it is suggested that TRPV1 triggers the release of SP and CGRP, which activate neurokinin-1 (NK1) and stimulate the sympathetic axis necessary for organ protection in endotoxemia [115]. 

Early studies investigated the role of afferent neurons in the development of LPS-induced intestinal obstruction in mice through the interaction between TRPV1 and CGRP [116]. Thus, afferent neurons that produce the neuropeptide CGRP and express TRPV1 play a crucial role in transmitting information from the periphery to the central nervous system during LPS-induced intestinal obstruction. Accordingly, blocking CGRP and TRPV1 may be a potential new strategy for treating endotoxin-induced intestinal obstruction. Another study demonstrated that peritoneal macrophages derived from TRPV1KO knockout mice exhibited reduced phagocytosis ability when stimulated with LPS [117]. Additionally, treatment with TRPV1 antagonist SB366791 decreased phagocytosis in attached wild-type macrophages. These findings suggest that TRPV1 can affect bacterial infection by enhancing the antibacterial function of macrophages.

TRPV4 has been shown to mediate the release of pro-inflammatory cytokines in LPS-stimulated mice [118]. LPS induced the production of cytokines IL-1B and IL-10 by macrophages. As such, in LPS-treated *TRPV4*^−/−^ knockout mice, IL-1B levels increased while IL-10 levels decreased. Therefore, TRPV4 may have a significant role in the cytokine response to bacterial infection-induced damage and may also be relevant to LPS-stimulated macrophage phagocytosis.

TRPM2-mediated Ca^2+^ influx induces the production of the chemokine CXCL2 in mouse monocytes, macrophages, and microglia under oxidative stress and LPS/IFN-γ stimulation [39]. It is worth noting that CXCL2 and inducible nitric oxide synthase were reduced in *TRPM2* knockout mice. The results indicate that TRPM2, which is expressed in macrophages and microglia, worsens peripheral and spinal nociceptive inflammatory responses and contributes to the development of inflammatory and neuropathic pain. TRPM2 plays a crucial role in CXCL2 secretion and the recruitment of peripheral and spinal immune cells, as well as the excitability and pathological increase in Ca^2+^ in damaged neurons, which exacerbate pathological pain. 

In mice, TRPM7 was found to mediate LPS-induced Ca^2+^ influx into macrophages. It regulates TLR4 endocytosis and stimulates the secretion of inflammatory cytokines associated with sepsis [119]. *TRPM7*-deficient macrophages cannot produce IL-1B and other important pro-inflammatory cytokines. Consequently, mice with *TRPM7* deletion are protected from LPS-induced peritonitis. These findings highlight the significance of Ca^2+^ signaling in macrophage activation and identify TRPM7 as a key component of TLR4 pathway. However, the molecular mechanism responsible for LPS-dependent TRPM7 activation remains unclear.

In *TRPC1*^−/−^-deficient mice, the secretion of IL-1B and IL-18 by macrophages was found to increase after LPS injection, regardless of caspase-1 activation [120]. Additionally, caspase-11 regulates the secretion of IL-1B and IL-18 both at the level of cytokine maturation through caspase-1 and extracellular release through TRPC1. Caspase-11 regulates inflammatory responses and cell death by controlling IL-1B secretion, enhancing caspase-1 activation, and inducing caspase-1-independent pyroptosis [121]. These findings suggest that TRPC1 plays a role in caspase-11-dependent IL-1B secretion and caspase-11-independent IL-18 secretion, indicating a potentially wider role for TRPC1 in regulating the immune response during bacterial infection.

Administering a Pyr10 inhibitor to block *TRPC3* channel alleviated the development of LPS-induced sepsis in mice by reducing the activation of several pro-inflammatory genes, including PTGS2, TNFA, IL-1B, and IL-6 [107]. These results suggest a protective role for TRPC3 in bacterial infections. Additionally, another study demonstrated that TRPC5 and TRPC4 can form a functional complex, promoting the accumulation of peritoneal leukocytes and the release of inflammatory mediators in mice [122]. In Trx+LPS model, mice were initially treated with *E. coli*-derived thioredoxin (Trx), which activates TRPC5, followed by LPS injection. This resulted in organ dysfunction-related mortality in mice, accompanied by a decrease in leukocyte accumulation, upregulation of IL-6 and IL-10 cytokine release into the peritoneum, and impaired phagocytosis mediated by peritoneal macrophages. However, dual blocking of TRPC4/TRPC5 using ML204 inhibitor with pre-injection of LPS and Trx (Trx+LPS+ML204 model) further increased mouse mortality and hypothermia, while maintaining macrophage phagocytosis. These findings suggest that the effects of bacterial Trx in combination with LPS may be mediated by TRPC4 and TRPC5, reflecting a possible mechanism of bacterial virulence and the pathophysiological role of these channels in sepsis.

Studies on LPS-induced sepsis models demonstrate that various TRP channels can detect harmful compounds and pathogens and induce the production of pro-inflammatory cytokines. There is evidence of a beneficial synergy between TRPA1, TRPV1, and TLR4 that enhances sensory and immune functions to eliminate bacterial endotoxins. Novel anti-inflammatory effects of TRPV1 in LPS-induced sepsis are also highlighted, wherein human models of TNBS- and DSS-induced colitis and IBD report predominantly pro-inflammatory characteristics of this channel. Furthermore, it is worth noting the role of TRP channels, specifically TRPM2, TRPM7, TRPC4, and TRPC5, in activating macrophages and regulating the secretion of inflammatory mediators. However, further studies are required to obtain more information on the effects and molecular mechanisms related to other TRP channels. Additionally, it is important to confirm or refute the already discovered effects in other models (Figure 1, Table 4).

#### 4.4.2. Cecal Ligation and Puncture

Cecal ligation and puncture (CLP) is a standard animal model for abdominal sepsis, which reflects the clinical course of intraperitoneal abscess and polymicrobial peritonitis [123]. The model allows to reproduce the activation of pro- and anti-inflammatory immune responses, early and late hypodynamic phases, multiple organ dysfunction, hypothermia, metabolic changes and cytokine response kinetics observed in humans. CLP also affects colonic tight junctions, causing changes in the cellular localization of claudins 1, 3, 4, 5, 8 and activation of claudin 2 [97]. There is also a similar model (colon ascendens stent peritonitis (CASP)), which reproduces the clinical situation of polymicrobial acute diffuse peritonitis with systemic inflammation and production of cytokines such as TNF-α, IL-1, IL-12, IL-18, IFN-γ, KC/GRO-α and MCP-1 [124]. However, CASP is not as commonly used as CLP and is more difficult to obtain because the biphasic immune response is not reproducible in sepsis [125]. To date, no studies have examined the possible effects of TRP channels in the CASP model. 

In the CLP model, TRPV1 knockout in mice was found to accelerate the onset of SIRS and enhance local inflammation [117]. Liver samples obtained from CLP *TRPV1KO* knockout mice showed an increase in bacterial rRNA compared to wild-type animals, indicating increased bacterial colonization of the abdomen. At the same time, a decrease in viable macrophages and MCP-1 and MIP-1β formation, as well as an increase in the levels of TNF-α, IL-10 and IL-6 in CLP *TRPV1KO* knockout mice, may contribute to immune suppression by deactivating macrophages and limiting the production of pro-inflammatory mediators. The impaired immune response may facilitate bacterial replication, thereby increasing the likelihood of severe infection. Based on the results obtained, TRPV1 appears to provide protection against immune and inflammatory reactions in the abdominal cavity, as well as subsequent SIRS. However, the mechanisms by which TRPV1 exerts this protective effect and its impact on SIRS are not yet fully understood. 

It has been reported that aged *Trpv1*^−/−^ knockout mice had a reduced ability to resist polymicrobial sepsis in the CLP model [126]. A similar result was obtained for aged animals that were administered the selective TRPV1 antagonist AMG517. The data suggest that the role of TRPV1 in systemic inflammation changes from anti-inflammatory to pro-inflammatory with aging. This shift may be due to changes in TRPV1 suppressive control of TNF-α production, among other factors. Therapy with TRPV1 antagonists in aged patients may potentially suppress the systemic inflammatory response, thereby reducing their resistance to bacterial infection and sepsis.

TRPV4 has been reported to be involved in the hyperinflammatory response and mortality associated with sepsis [127]. Thus, the highly selective inhibitors GSK2193874 and HC067047 were used to block TRPV4 in mice with CLP, resulting in reduced mortality by decreasing the levels of pro-inflammatory cytokines TNF-α, IL-1 and IL-6, and subsequently maintaining intracellular Ca^2+^ influx. These results suggest that TRPV4 antagonists may have therapeutic value in the treatment of sepsis.

TRPA1 activation has been demonstrated to play a protective role in a mouse model of CLP by regulating the anti-inflammatory response [128]. Vagus nerve TRPA1 signaling inhibited cytokine release, inducing hypothermia and reducing mortality due to infection. Additionally, it has been shown that TRPA1 can mediate sepsis-induced immunosuppression in response to IL-1B. Further research is required to assess the potential importance of molecular pathways in vagal IL-1B/TRPA1 signaling and their combined role in immune regulation during sepsis.

In a mouse model of CLP, TRPM2 channel has been demonstrated to be crucial in controlling invading bacteria by regulating the production of pro-inflammatory cytokines and the expression of heme oxygenase 1 (HO-1) [129]. HO-1 is an enzyme that protects against damage during inflammation and microbial sepsis by enhancing bacterial clearance [130]. It is suggested that TRPM2 involvement in eliminating bacterial agents may be mediated by HO-1. This finding correlates with TRPM2-mediated Ca^2+^ influx regulating HO-1 expression in macrophages. Additionally, in bone marrow-derived macrophages from both wild-type mice and wild-type mice with CLP, HO-1 reduced the bacterial load. Disrupting TRPM2 decreased HO-1 expression and increased the bacterial load. Furthermore, *TRPM2*^−/−^ mice have been reported to experience increased mortality, suggesting the potential for targeting TRPM2 channel as an immunoadjuvant therapy for sepsis in humans.

Another channel, TRPM4, also developed a protective effect in a mouse model of CLP [131]. Ca^2+^ influx is crucial for the functioning of all hematopoietic cells. However, TRPM4 deficiency resulted in the impaired Ca^2+^ flow in macrophages, inhibiting their phagocytic activity, thereby leading to excess bacterial growth. Additionally, the lack of TRPM4 compromised the peritoneal macrophage population, elevated systemic levels of Ly6C^+^ inflammatory monocytes, and increased the production of pro-inflammatory cytokines. These findings demonstrate the important role of TRPM4 in regulating Ca^2+^ influx in immune cells and the consequent response to infection. Nevertheless, the mechanisms responsible for Ca^2+^ regulation in immune cells have not been clarified yet.

Limited studies on CLP models suggest that TRP channels, specifically TRPV1, TRPA1, and TRPM4, provide protection against immune and inflammatory responses (Table 4). However, the mechanisms by which these TRP channels exert their protective effects and their influence on SIRS are not yet fully understood. In the CLP model, similar to the LPS-induced sepsis model, TRPV1 demonstrates anti-inflammatory effects, which contradicts the findings for human models of colitis and IBD. Additionally, it has been suggested that TRPV4 and TRPM2 antagonists may have therapeutic potential in treating sepsis. Further research is necessary to reconcile these discrepancies and enhance comprehension of the molecular mechanisms underlying TRP-mediated pathogen recognition and immune response.

**Table 4:** Main effects of TRP channels in animal sepsis models.

TRP Channel	Model	Object	Confirmed and/or Expected Effects	References
TRPA1	LPS-induced sepsis model	mice	Recognition of *E. coli* LPS independently of TLR4, possible role in the initial response to pathogens, acute neurogenic inflammation and pain	[106]
	CLP model	mice	Protective role, regulation of the anti-inflammatory response	[128]
TRPV1	LPS-induced sepsis model	mice	LPS sensor	[112]
	LPS-induced peritonitis	mice	Visceral pain	[114]
	LPS-induced peritonitis	mice	Anti-inflammatory effect on the spleen, protective effect in sepsis	[115]
	LPS-induced sepsis model	mice	Enhancing the antibacterial function of macrophages	[117]
	CLP model	mice	Protective role in bacterial infection and inflammation	[117]
TRPV4	LPS-induced sepsis model	mice	Regulation of pro-inflammatory cytokine release and LPS-stimulated macrophage phagocytosis	[118]
	CLP model	mice	Hyperinflammatory response and mortality associated with sepsis	[127]
TRPM2	LPS-induced sepsis model	mice	Stimulation of the chemokine CXCL2 production, inflammatory and neuropathic pain	[39]
	CLP model	mice	Regulation of pro-inflammatory cytokine production and expression of HO-1	[129]
TRPM4	CLP model	mice	Protective effect	[131]
TRPM7	LPS-induced sepsis model	mice	Regulation of TLR4 endocytosis and stimulation of inflammatory cytokine secretion associated with sepsis	[119]
TRPC1	LPS-induced sepsis model	mice	Regulation of caspase-11-dependent IL-1B secretion and caspase-11-independent IL-18 secretion	[120,121]
TRPC3	LPS-induced sepsis model	mice	Protective role in bacterial infections	[107]
TRPC4/TRPC5 functional complex	Trx+LPS model	mice	Promotion of peritoneal leukocyte accumulation and inflammatory mediator release	[122]

## 5. Concluding Remarks

This review has provided valuable insights into the complex roles of TRP channels in both IBD and sepsis. In the context of IBD, a number of TRP channels, including TRPV1, TRPV2, TRPA1, TRPM2 and TRPV4, have emerged as pivotal mediators of inflammatory responses, demonstrating pro-inflammatory and/or anti-inflammatory effects depending on the animal model used. primarily due to the differences in experimental conditions and the diverse effects of colitis-inducing substances on the severity of colitis and intestinal inflammation. The data obtained are generally consistent with the limited results in human epithelial cell lines and human intestinal samples of CD and UC patients.

In sepsis, particularly in models such as LPS-induced sepsis and CLP, TRP channels exhibit diverse functions in modulating immune and inflammatory processes. For instance, TRPC3 blockade has shown promise as a therapeutic intervention in mitigating sepsis development, while the interaction between TRPC5 and TRPC4 contributes to leukocyte accumulation and the release of inflammatory mediators. Similarly, investigations using the CLP model have unveiled the intricate effects of TRPV1 deletion or activation on local inflammation and SIRS, underscoring the multifaceted nature of TRP involvement in sepsis pathogenesis. Furthermore, TRPA1 signaling in vagal afferents has been implicated in inhibiting cytokine release, inducing hypothermia, and reducing mortality from infection, highlighting its protective role. Additionally, TRPM2 and TRPM4 channels have been shown to enhance bacterial clearance and regulate inflammatory responses in CLP-induced sepsis. 

In general, the diverse roles of TRP in sepsis and IBD should be highlighted, which are likely the result of a complex interplay between multiple factors, including the different mechanisms of activation in colitis and sepsis, the localization of TRP channels (immune cells, neurons, etc.), the microenvironment, the degree of colon inflammation, and the organism type (human or animal model). Furthermore, to overcome the existing limitations, future studies of TRP involving specific models of colitis or sepsis should consider the following aspects: different models may demonstrate inconsistent data depending on the experimental protocol, inductor substance, object, agonists/antagonists applied; the obtained results in view of the clinical correlation shall be interpreted cautiously; it is important to take into account the potential synergistic effects of different TRP channels and the ambiguous role (pro-inflammatory and/or anti-inflammatory) that they may play depending on the model used.

Despite the progress achieved, numerous challenges and unanswered questions persist, necessitating further research to elucidate the intricate molecular pathways underlying TRP-mediated immune modulation and pathogen recognition. Addressing these gaps not only advances our comprehension and management of sepsis but also sheds light on the broader implications of TRP channels in inflammatory disorders such as IBD. Future investigations are warranted to explore the therapeutic potential of targeting specific TRP channels in the management of sepsis and related inflammatory conditions, thereby paving the way for novel therapeutic interventions in critical care medicine.

## Figures and Tables

**Table 1:** The role of TRP channels in IBD.

TRP Channel	Sample Source/Cell Line	Confirmed and/or Expected Effects	References
TRPA1	Samples from patients with active and inactive forms of CD and UC	Reduced expression of several pro-inflammatory neuropeptides, cytokines, and chemokines, a decrease in inflammation	[28]
	Colon tissue samples from patients with active forms of CD and UC	Reduced severity of chronic T-cell-mediated colitis, protective role in inflammation	[55]
TRPV1	Colonic epithelium samples from patients with active forms of CD and UC	Colitogenic responses of CD4^+^ T cells and intestinal inflammation	[56,57]
	Samples from patients with UC in remission	Abdominal pain and visceral hypersensitivity	[60]
TRPV4	Colon biopsies of patients with CD and UC	Elevated intracellular Ca^2+^ concentrations, release of chemokines, and chronic inflammation	[61]
	Human intestinal epithelial cell lines Caco-2 and T84	Pro-inflammatory response, chronic inflammation	[62]
	Mucosal epithelial cells of patients with UC	Increased inflammation	[58]

**Table 2:** Main effects of TRP in animal colitis models.

TRP Channel	Model	Object	Confirmed and/or Expected Effects	References
TRPA1	TNBS-induced colitis model	mice	Increased inflammation and fibrosis, acute pain	[69]
	TNBS-induced colitis model	rat	Visceral hypersensitivity (a synergistic effect with TRPV1)	[70]
	DSS-induced colitis model	mice	Protective role in colitis	[28]
	DSS-induced colitis model	mice	Visceral hypersensitivity mediated by TRPA1 agonist AITC	[81]
	OM-induced colitis	mice	Increased mRNA levels of various neuropeptides and mediators associated with pain and inflammation	[83]
	AOM/DSS model	mice	Increased apoptotic and oxidative effects in colon cancer cells (combined effect of TRPA1, TRPM2 and TRPV1)	[85]
TRPV1	TNBS-induced colitis model	rat	Visceral hypersensitivity (a synergistic effect with TRPV2)	[71]
	TNBS-induced colitis model	rat	Visceral hypersensitivity (a synergistic effect with TRPA1)	[70]
	DSS-induced colitis model	mice	Increased inflammation, increased release of CGRP and SP, visceral hypersensitivity and pain-related behavior	[30,31,78,80]
	Oxazolone-induced colitis	mice	Excessive neutrophil accumulation	[82]
	DNBS-induced colitis model	mice	Modulating of sensory pathways involved in colonic inflammation, possible protective effect	[84]
	AOM/DSS model	mice	Increased apoptotic and oxidative effects in colon cancer cells (combined effect of TRPA1, TRPM2 and TRPV1)	[85]
TRPV2	TNBS-induced colitis model	rat	Visceral hypersensitivity (a synergistic effect with TRPV1)	[71]
	DSS-induced colitis model	mice	Increased inflammation	[35,79]
TRPV4	TNBS-induced colitis model	mice	Intestinal inflammation and colitis-associated pain	[61]
	DSS-induced colitis model	mice	Pro-inflammatory effects	[36]
TRPM2	TNBS-induced colitis model	rat	Increased visceromotor reflexes caused by balloon pressure, visceral hypersensitivity	[68]
	DSS-induced colitis model	mice	Progression of colitis through its possible implication in oxidative stress signaling	[73]
	AOM/DSS model	mice	Increased apoptotic and oxidative effects in colon cancer cells (combined effect of TRPA1, TRPM2 and TRPV1)	[85]
TRPM3	DSS-induced colitis model	mice	Perception of noxious stimuli in colitis, colonic hypersensitivity	[74]
TRPM8	TNBS-induced colitis model	mice	Visceral hyperalgesia	[75]
	DSS-induced colitis model	mice	Anti-inflammatory effects	[76]

**Table 3:** TRP effects in human cell lines.

TRP Channel	Human Cell Line	Confirmed and/or Expected Effects	References
TRPV1	HEK293T cells	LPS sensor, impaired membrane stability, pain and inflammation associated with bacterial infection	[103]
TRPM2	Human THP-1 monocytes	Regulation of LPS-induced production of pro-inflammatory cytokines IL-6, IL-8, IL-10, and TNF-α	[38]
TRPM3	HEK293T cells	LPS sensor, impaired membrane stability	[103]
TRPM8	HEK293T cells	LPS sensor, impaired membrane stability	[103]
TRPC3	HEK293 cells (HEK-TLR4 cells)	Significant increase in the expression levels of pro-inflammatory genes PTGS2, TNFA and IL-12B, immune response to bacterial endotoxins	[107]

## Data Availability

No new data were created or analyzed in this study. Data sharing is not applicable to this article.

## Abbreviations

TRP	Transient receptor potential channel
IBD	Inflammatory bowel disease
CD	Crohn’s disease
UC	Ulcerative colitis
GI	Gastrointestinal tract
EC	Epithelial cell
DC	Dendritic cell
Th	T-helper cells
TLR4	Toll-like receptor 4
Tregs	Regulatory T cells
IgA	Immunoglobulin A
CGRP	Calcitonin Gene-Related Peptide
SP	Substance P
NKA	Neurokinin A
NKB	Neurokinin B
IL	Interleukin
TNF	Tumor necrosis factor
IFN	Interferon
NGF	Nerve growth factor
PAR2	Protease-activated receptor 2
APC	Antigen-presenting cell
TGF-β	Transforming growth factor beta
PDE4	Phosphodiesterase-4 inhibitor
JAK	Janus Kinase Inhibitor
SMAD7	Mothers against decapentaplegic homolog 7
S1P	Sphingosine-1-phosphate receptor 1
4α-PDD	4α-phorbol-12,13-didecanoate
siRNA	Small interfering RNA
TNBS	2,4,6-Trinitrobenzene sulfonic acid
DRG	Dorsal root ganglion
NG	Nodose ganglion
DSS	Dextran sulfate sodium
WDR	Wide dynamic range
AITC	Allyl isothiocyanate
OM	Oil of mustard
DNBS	Dinitrobenzene sulfonic acid
AOM	Azoxymethane
SIRS	Systemic inflammatory response syndrome
PRRs	Pattern recognition receptors
PAMPs	Pathogen-associated molecular patterns
DAMPs	Damage-associated molecular patterns
NLRs	NOD-like receptors
RLRs	RIG-I-like receptors
MDSC	Myeloid-derived suppressor cell
LPS	Lipopolysaccharide
MAPK	Mitogen-activated protein kinase
NF-kB	Nuclear factor kappa-light-chain-enhancer of activated B cells
AP-1	Activating protein-1
IRF-3	Interferon regulatory factor 3
CASP	Colon ascendens stent peritonitis
CLP	Cecal ligation and puncture
HO-1	Heme oxygenase 1

## References

[1] Zhang M., Ma Y., Ye X., Zhang N., Pan L., Wang B. (2023). TRP (transient receptor potential) ion channel family: Structures, biological functions and therapeutic interventions for diseases. Signal Transduct. Target. Ther..

[2] Himmel N.J., Cox D.N. (2020). Transient receptor potential channels: Current perspectives on evolution, structure, function and nomenclature. Proc. R. Soc. B Biol. Sci..

[3] Cao E. (2020). Structural mechanisms of transient receptor potential ion channels. J. Gen. Physiol..

[4] Fine M., Li X., Dang S. (2019). Structural insights into group II TRP channels. Cell Calcium.

[5] Mickle A.D., Shepherd A.J., Mohapatra D.P. (2016). Nociceptive TRP Channels: Sensory Detectors and Transducers in Multiple Pain Pathologies. Pharmaceuticals.

[6] Skerratt S. (2017). Recent Progress in the Discovery and Development of TRPA1 Modulators. Prog. Med. Chem..

[7] Abbas M.A. (2020). Modulation of TRPV1 channel function by natural products in the treatment of pain. Chem. Interactions.

[8] Jeong K.-Y., Seong J. (2014). Neonatal capsaicin treatment in rats affects TRPV1-related noxious heat sensation and circadian body temperature rhythm. J. Neurol. Sci..

[9] Martinez G.Q., Gordon S.E. (2019). Multimerization of Homo sapiens TRPA1 ion channel cytoplasmic domains. PLoS ONE.

[10] Csekő K., Beckers B., Keszthelyi D., Helyes Z. (2019). Role of TRPV1 and TRPA1 Ion Channels in Inflammatory Bowel Diseases: Potential Therapeutic Targets?. Pharmaceuticals.

[11] Vergnolle N. (2014). TRPV4: New therapeutic target for inflammatory bowel diseases. Biochem. Pharmacol..

[12] Weinstock J.V. (2015). Substance P and the regulation of inflammation in infections and inflammatory bowel disease. Acta Physiol..

[13] Alaimo A., Rubert J. (2019). The Pivotal Role of TRP Channels in Homeostasis and Diseases throughout the Gastrointestinal Tract. Int. J. Mol. Sci..

[14] Zhang L., Simonsen C., Zimova L., Wang K., Moparthi L., Gaudet R., Ekoff M., Nilsson G., Hellmich U.A., Vlachova V. (2022). Cannabinoid non-cannabidiol site modulation of TRPV2 structure and function. Nat. Commun..

[15] Su W., Qiao X., Wang W., He S., Liang K., Hong X. (2023). TRPV3: Structure, Diseases and Modulators. Molecules.

[16] Zuo X., Ling J.X., Xu G.-Y., Gu J.G. (2013). Operant Behavioral Responses to Orofacial Cold Stimuli in Rats with Chronic Constrictive Trigeminal Nerve Injury: Effects of Menthol and Capsazepine. Mol. Pain.

[17] Tan C.-H., McNaughton P.A. (2016). The TRPM2 ion channel is required for sensitivity to warmth. Nature.

[18] Jang Y., Cho P.S., Yang Y.D., Hwang S.W. (2018). Nociceptive Roles of TRPM2 Ion Channel in Pathologic Pain. Mol. Neurobiol..

[19] Su S., Yudin Y., Kim N., Tao Y.-X., Rohacs T. (2021). TRPM3 Channels Play Roles in Heat Hypersensitivity and Spontaneous Pain after Nerve Injury. J. Neurosci..

[20] Cortright D.N., Szallasi A. (2009). TRP Channels and Pain. Curr. Pharm. Des..

[21] Klimas J., Gorfinkel L., Fairbairn N., Amato L., Ahamad K., Nolan S., Simel D.L., Wood E. (2019). Strategies to Identify Patient Risks of Prescription Opioid Addiction When Initiating Opioids for Pain. JAMA Netw. Open.

[22] Hung C.-Y., Tan C.-H. (2018). TRP Channels in Nociception and Pathological Pain. Advances in Pain Research: Mechanisms and Modulation of Chronic Pain.

[23] Anderson E.M., Jenkins A.C., Caudle R.M., Neubert J.K. (2014). The Effects of a Co-Application of Menthol and Capsaicin on Nociceptive Behaviors of the Rat on the Operant Orofacial Pain Assessment Device. PLoS ONE.

[24] Honore P., Chandran P., Hernandez G., Gauvin D.M., Mikusa J.P., Zhong C., Joshi S.K., Ghilardi J.R., Sevcik M.A., Fryer R.M. (2009). Repeated dosing of ABT-102, a potent and selective TRPV1 antagonist, enhances TRPV1-mediated analgesic activity in rodents, but attenuates antagonist-induced hyperthermia. Pain.

[25] Miller F., Björnsson M., Svensson O., Karlsten R. (2014). Experiences with an adaptive design for a dose-finding study in patients with osteoarthritis. Contemp. Clin. Trials.

[26] Song P., Armstrong C. (2019). Novel therapeutic approach with PAC -14028 cream, a TRPV 1 antagonist, for patients with mild-to-moderate atopic dermatitis. Br. J. Dermatol..

[27] Shaabani S., Huizinga H.P.S., Butera R., Kouchi A., Guzik K., Magieramularz K., Holak T.A., Domling A. (2018). A patent review on PD-1/PD-L1 antagonists: Small molecules, peptides, and macrocycles (2015–2018). Expert Opin. Ther. Pat..

[28] Kun J., Szitter I., Kemény Á., Perkecz A., Kereskai L., Pohóczky K., Vincze A., Gódi S., Szabó I., Szolcsányi J. (2014). Upregulation of the Transient Receptor Potential Ankyrin 1 Ion Channel in the Inflamed Human and Mouse Colon and Its Protective Roles. PLoS ONE.

[29] Xiao T., Sun M., Kang J., Zhao C. (2022). Transient Receptor Potential Vanilloid1 (TRPV1) Channel Opens Sesame of T Cell Responses and T Cell-Mediated Inflammatory Diseases. Front. Immunol..

[30] Duo L., Wu T., Ke Z., Hu L., Wang C., Teng G., Zhang W., Wang W., Ge Q., Yang Y. (2020). Gain of Function of Ion Channel TRPV1 Exacerbates Experimental Colitis by Promoting Dendritic Cell Activation. Mol. Ther. Nucleic Acids.

[31] Lapointe T.K., Basso L., Iftinca M.C., Flynn R., Chapman K., Dietrich G., Vergnolle N., Altier C., Abdullah N., Defaye M. (2015). TRPV1 sensitization mediates postinflammatory visceral pain following acute colitis. Am. J. Physiol. Liver Physiol..

[32] Melnick C., Kaviany M. (2018). Thermal actuation in TRPV1: Role of embedded lipids and intracellular domains. J. Theor. Biol..

[33] Lapointe T.K., Altier C. (2011). The role of TRPA1 in visceral inflammation and pain. Channels.

[34] de Araujo D.S.M., Nassini R., Geppetti P., De Logu F. (2020). TRPA1 as a therapeutic target for nociceptive pain. Expert Opin. Ther. Targets.

[35] Issa C.M., Hambly B.D., Wang Y., Maleki S., Wang W., Fei J., Bao S. (2014). TRPV2 in the Development of Experimental Colitis. Scand. J. Immunol..

[36] Matsumoto K., Yamaba R., Inoue K., Utsumi D., Tsukahara T., Amagase K., Tominaga M., Kato S. (2017). Transient receptor potential vanilloid 4 channel regulates vascular endothelial permeability during colonic inflammation in dextran sulphate sodium-induced murine colitis. Br. J. Pharmacol..

[37] Poole D.P., Amadesi S., Veldhuis N.A., Abogadie F.C., Lieu T., Darby W., Liedtke W., Lew M.J., McIntyre P., Bunnett N.W. (2013). Protease-activated Receptor 2 (PAR2) Protein and Transient Receptor Potential Vanilloid 4 (TRPV4) Protein Coupling Is Required for Sustained Inflammatory Signaling*. J. Biol. Chem..

[38] Wehrhahn J., Kraft R., Harteneck C., Hauschildt S. (2010). Transient receptor potential melastatin 2 is required for lipopolysaccharide-induced cytokine production in human monocytes. J. Immunol..

[39] Haraguchi K., Kawamoto A., Isami K., Maeda S., Kusano A., Asakura K., Shirakawa H., Mori Y., Nakagawa T., Kaneko S. (2012). TRPM2 Contributes to Inflammatory and Neuropathic Pain through the Aggravation of Pronociceptive Inflammatory Responses in Mice. J. Neurosci..

[40] Bruner L.P., White A.M., Proksell S. (2023). Inflammatory Bowel Disease. Prim. Care Clin. Off. Pr..

[41] Cockburn E., Kamal S., Chan A., Rao V., Liu T., Huang J.Y., Segal J.P. (2023). Crohn’s disease: An update. Clin. Med..

[42] Le Berre C., Honap S., Peyrin-Biroulet L. (2023). Ulcerative colitis. Lancet.

[43] Saez A., Herrero-Fernandez B., Gomez-Bris R., Sánchez-Martinez H., Gonzalez-Granado J.M. (2023). Pathophysiology of Inflammatory Bowel Disease: Innate Immune System. Int. J. Mol. Sci..

[44] Inoue R., Kurahara L.-H., Hiraishi K. (2018). TRP channels in cardiac and intestinal fibrosis. Semin. Cell Dev. Biol..

[45] Yun S.-M., Kim S.-H., Kim E.-H. (2019). The Molecular Mechanism of Transforming Growth Factor-β Signaling for Intestinal Fibrosis: A Mini-Review. Front. Pharmacol..

[46] Liu J., Di B., Xu L.-L. (2023). Recent advances in the treatment of IBD: Targets, mechanisms and related therapies. Cytokine Growth Factor. Rev..

[47] Wang Q., Chen F., Peng Y., Yi X., He Y., Shi Y. (2023). Research Progress of Interleukin-27 in Inflammatory Bowel Disease. Inflamm. Bowel Dis..

[48] Flynn S., Eisenstein S. (2019). Inflammatory Bowel Disease Presentation and Diagnosis. Surg. Clin. North. Am..

[49] Zielińska M., Jarmuż A., Wasilewski A., Sałaga M., Fichna J. (2015). Role of Transient Receptor Potential Channels in Intestinal Inflammation and Visceral Pain. Inflamm. Bowel Dis..

[50] Chen Y., Mu J., Zhu M., Mukherjee A., Zhang H. (2020). Transient Receptor Potential Channels and Inflammatory Bowel Disease. Front. Immunol..

[51] Engel M.A., Leffler A., Niedermirtl F., Babes A., Zimmermann K., Filipović M.R., Izydorczyk I., Eberhardt M., Kichko T.I., Mueller–Tribbensee S.M. (2011). TRPA1 and Substance P Mediate Colitis in Mice. Gastroenterology.

[52] Du Y., Chen J., Shen L., Wang B. (2022). TRP channels in inflammatory bowel disease: Potential therapeutic targets. Biochem. Pharmacol..

[53] Fischer M.J., Edwardson J.M. (2014). V2A2lidating TRP channel heteromers. Temperature.

[54] Bertin S., Aoki-Nonaka Y., Lee J., de Jong P.R., Kim P., Han T., Yu T., To K., Takahashi N., Boland B.S. (2016). The TRPA1 ion channel is expressed in CD4+ T cells and restrains T-cell-mediated colitis through inhibition of TRPV1. Gut.

[55] Engel M.A., Becker C., Reeh P.W., Neurath M.F. (2011). Role of sensory neurons in colitis: Increasing evidence for a neuroimmune link in the gut. Inflamm. Bowel Dis..

[56] Bertin S., Aoki-Nonaka Y., de Jong P.R., Nohara L.L., Xu H., Stanwood S.R., Srikanth S., Lee J., To K., Abramson L. (2014). The ion channel TRPV1 regulates the activation and proinflammatory properties of CD4+ T cells. Nat. Immunol..

[57] Luo C., Wang Z., Mu J., Zhu M., Zhen Y., Zhang H. (2017). Upregulation of the transient receptor potential vanilloid 1 in colonic epithelium of patients with active inflammatory bowel disease. Int. J. Clin. Exp. Pathol..

[58] Rizopoulos T., Papadaki-Petrou H., Assimakopoulou M. (2018). Expression Profiling of the Transient Receptor Potential Vanilloid (TRPV) Channels 1, 2, 3 and 4 in Mucosal Epithelium of Human Ulcerative Colitis. Cells.

[59] Keszthelyi D., Troost F., Jonkers D., Helyes Z., Hamer H., Ludidi S., Vanhoutvin S., Venema K., Dekker J., Szolcsányi J. (2013). Alterations in mucosal neuropeptides in patients with irritable bowel syndrome and ulcerative colitis in remission: A role in pain symptom generation?. Eur. J. Pain.

[60] Akbar A., Yiangou Y., Facer P., Brydon W.G., Walters J.R.F., Anand P., Ghosh S. (2010). Expression of the TRPV1 receptor differs in quiescent inflammatory bowel disease with or without abdominal pain. Gut.

[61] Fichna J., Mokrowiecka A., Cygankiewicz A.I., Zakrzewski P.K., Małecka-Panas E., Janecka A., Krajewska W.M., Storr M.A. (2012). Transient Receptor Potential Vanilloid 4 blockade protects against experimental colitis in mice: A new strategy for inflammatory bowel diseases treatment?. Neurogastroenterol. Motil..

[62] D’Aldebert E., Cenac N., Rousset P., Martin L., Rolland C., Chapman K., Selves J., Alric L., Vinel J., Vergnolle N. (2011). Transient Receptor Potential Vanilloid 4 Activated Inflammatory Signals by Intestinal Epithelial Cells and Colitis in Mice. Gastroenterology.

[63] Davis J., Burr A.R., Davis G.F., Birnbaumer L., Molkentin J.D. (2012). A TRPC6-Dependent Pathway for Myofibroblast Transdifferentiation and Wound Healing In Vivo. Dev. Cell.

[64] Kurahara L.H., Hiraishi K., Hu Y., Koga K., Onitsuka M., Doi M., Aoyagi K., Takedatsu H., Kojima D., Fujihara Y. (2017). Activation of Myofibroblast TRPA1 by Steroids and Pirfenidone Ameliorates Fibrosis in Experimental Crohn’s Disease. Cell. Mol. Gastroenterol. Hepatol..

[65] Hiraishi K., Kurahara L.-H., Sumiyoshi M., Hu Y.-P., Koga K., Onitsuka M., Kojima D., Yue L., Takedatsu H., Jian Y.-W. (2018). Daikenchuto (Da-Jian-Zhong-Tang) ameliorates intestinal fibrosis by activating myofibroblast transient receptor potential ankyrin 1 channel. World J. Gastroenterol..

[66] Bilsborough J.M., Fiorino M.F., Henkle B.W. (2020). Select animal models of colitis and their value in predicting clinical efficacy of biological therapies in ulcerative colitis. Expert Opin. Drug Discov..

[67] Antoniou E., Margonis G.A., Angelou A., Pikouli A., Argiri P., Karavokyros I., Papalois A., Pikoulis E. (2016). The TNBS-induced colitis animal model: An overview. Ann. Med. Surg..

[68] Matsumoto K., Takagi K., Kato A., Ishibashi T., Mori Y., Tashima K., Mitsumoto A., Kato S., Horie S. (2016). Role of transient receptor potential melastatin 2 (TRPM2) channels in visceral nociception and hypersensitivity. Exp. Neurol..

[69] Cattaruzza F., Johnson C., Leggit A., Grady E., Schenk A.K., Cevikbas F., Cedron W., Bondada S., Kirkwood R., Malone B. (2013). Transient receptor potential ankyrin 1 mediates chronic pancreatitis pain in mice. Am. J. Physiol. Liver Physiol..

[70] Vermeulen W., De Man Joris G., De Schepper Heiko U., Bult H., Moreels T.G., Pelckmans P.A., De Winter Benedicte Y. (2013). Role of TRPV1 and TRPA1 in visceral hypersensitivity to colorectal distension during experimental colitis in rats. Eur. J. Pharmacol..

[71] Matsumoto K., Sugimoto F., Mizuno T., Hayashi T., Okamura R., Nishioka T., Yasuda H., Horie S., Kido M.A., Kato S. (2022). Immunohistochemical characterization of transient receptor potential vanilloid types 2 and 1 in a trinitrobenzene sulfonic acid-induced rat colitis model with visceral hypersensitivity. Cell Tissue Res..

[72] Yang C., Merlin D. (2024). Unveiling Colitis: A Journey through the Dextran Sodium Sulfate-induced Model. Inflamm. Bowel Dis..

[73] Yamamoto S., Shimizu S., Kiyonaka S., Takahashi N., Wajima T., Hara Y., Negoro T., Hiroi T., Kiuchi Y., Okada T. (2008). TRPM2-mediated Ca^2+^ influx induces chemokine production in monocytes that aggravates inflammatory neutrophil infiltration. Nat. Med..

[74] King J.W., Bennett A.S.W., Wood H.M., Baker C.C., Alsaadi H., Topley M., Vanner S.A., Reed D.E., Lomax A.E. (2024). Expression and function of transient receptor potential melastatin 3 in the spinal afferent innervation of the mouse colon. Am. J. Physiol. Liver Physiol..

[75] Hosoya T., Matsumoto K., Tashima K., Nakamura H., Fujino H., Murayama T., Horie S. (2014). TRPM8 has a key role in experimental colitis-induced visceral hyperalgesia in mice. Neurogastroenterol. Motil..

[76] Ramachandran R., Hyun E., Zhao L., Lapointe T.K., Chapman K., Hirota C.L., Ghosh S., McKemy D.D., Vergnolle N., Beck P.L. (2013). TRPM8 activation attenuates inflammatory responses in mouse models of colitis. Proc. Natl. Acad. Sci. USA.

[77] Khalil M., Babes A., Lakra R., Försch S., Reeh P., Wirtz S., Becker C., Neurath M., Engel M. (2016). Transient receptor potential melastatin 8 ion channel in macrophages modulates colitis through a balance-shift in TNF-alpha and interleukin-10 production. Mucosal Immunol..

[78] Engel M.A., Khalil M., Mueller-Tribbensee S.M., Becker C., Neuhuber W.L., Neurath M.F., Reeh P.W. (2011). The proximodistal aggravation of colitis depends on substance P released from TRPV1-expressing sensory neurons. J. Gastroenterol..

[79] Santoni G., Farfariello V., Liberati S., Morelli M.B., Nabissi M., Santoni M., Amantini C. (2013). The role of transient receptor potential vanilloid type-2 ion channels in innate and adaptive immune responses. Front. Immunol..

[80] Utsumi D., Matsumoto K., Tsukahara T., Amagase K., Tominaga M., Kato S. (2018). Transient receptor potential vanilloid 1 and transient receptor potential ankyrin 1 contribute to the progression of colonic inflammation in dextran sulfate sodium-induced colitis in mice: Links to calcitonin gene-related peptide and substance P. J. Pharmacol. Sci..

[81] Mitrovic M., Shahbazian A., Bock E., Pabst M.A., Holzer P. (2010). Chemo-nociceptive signalling from the colon is enhanced by mild colitis and blocked by inhibition of transient receptor potential ankyrin 1 channels. Br. J. Pharmacol..

[82] Lee J., Yamamoto T., Kuramoto H., Kadowaki M. (2012). TRPV1 expressing extrinsic primary sensory neurons play a protective role in mouse oxazolone-induced colitis. Auton. Neurosci..

[83] Kimball E.S., Prouty S.P., Pavlick K.P., Wallace N.H., Schneider C.R., Hornby P.J. (2007). Stimulation of neuronal receptors, neuropeptides and cytokines during experimental oil of mustard colitis. Neurogastroenterol. Motil..

[84] Massa F., Sibaev A., Marsicano G., Blaudzun H., Storr M., Lutz B. (2005). Vanilloid receptor (TRPV1)-deficient mice show increased susceptibility to dinitrobenzene sulfonic acid induced colitis. J. Mol. Med..

[85] Kaya M.M., Kaya I., Nazıroğlu M. (2022). Transient receptor potential channel stimulation induced oxidative stress and apoptosis in the colon of mice with colitis-associated colon cancer: Modulator role of *Sambucus ebulus* L. Mol. Biol. Rep..

[86] Srzić I., Adam V.N., Pejak D.T. (2022). Sepsis definition: What’s new in the Treatment Guidelines. Acta Clin. Croat..

[87] Santajit S., Indrawattana N. (2016). Mechanisms of Antimicrobial Resistance in ESKAPE Pathogens. BioMed Res. Int..

[88] Minasyan H. (2019). Sepsis: Mechanisms of bacterial injury to the patient. Scand. J. Trauma, Resusc. Emerg. Med..

[89] van der Poll T., van de Veerdonk F.L., Scicluna B.P., Netea M.G. (2017). The immunopathology of sepsis and potential therapeutic targets. Nat. Rev. Immunol..

[90] Li D., Wu M. (2021). Pattern recognition receptors in health and diseases. Signal Transduct. Target. Ther..

[91] Liu D., Huang S.-Y., Sun J.-H., Zhang H.-C., Cai Q.-L., Gao C., Li L., Cao J., Xu F., Zhou Y. (2022). Sepsis-induced immunosuppression: Mechanisms, diagnosis and current treatment options. Mil. Med. Res..

[92] Hotchkiss R.S., Monneret G., Payen D. (2013). Sepsis-induced immunosuppression: From cellular dysfunctions to immunotherapy. Nat. Rev. Immunol..

[93] Delano M.J., Ward P.A. (2016). Sepsis-induced immune dysfunction: Can immune therapies reduce mortality?. J. Clin. Investig..

[94] Payen D. (2020). The gut as a hidden source of sepsis. Minerva Anestesiol..

[95] Fay K.T., Ford M.L., Coopersmith C.M. (2017). The intestinal microenvironment in sepsis. Biochim. et Biophys. Acta (BBA) Mol. Basis Dis..

[96] Boldingh Q.J., de Vries F.E., Boermeester M.A. (2017). Abdominal sepsis. Curr. Opin. Crit. Care.

[97] Yoseph B.P., Klingensmith N.J., Liang Z., Breed E.R., Burd E.M., Mittal R., Dominguez J.A., Petrie B., Ford M.L., Coopersmith C.M. (2016). Mechanisms of Intestinal Barrier Dysfunction in Sepsis. Shock.

[98] Shen L. (2012). Tight junctions on the move: Molecular mechanisms for epithelial barrier regulation. Ann. New York Acad. Sci..

[99] Thome J.J., Yudanin N., Ohmura Y., Kubota M., Grinshpun B., Sathaliyawala T., Kato T., Lerner H., Shen Y., Farber D.L. (2014). Spatial Map of Human T Cell Compartmentalization and Maintenance over Decades of Life. Cell.

[100] Dasgupta S., Erturk-Hasdemir D., Ochoa-Reparaz J., Reinecker H.-C., Kasper D.L. (2014). Plasmacytoid Dendritic Cells Mediate Anti-inflammatory Responses to a Gut Commensal Molecule via Both Innate and Adaptive Mechanisms. Cell Host Microbe.

[101] Suzuki K. (2020). Diversified IgA-Bacteria Interaction in Gut Homeostasis. Adv. Exp. Med. Biol..

[102] Wiersinga W.J., van der Poll T. (2022). Immunopathophysiology of human sepsis. EBioMedicine.

[103] Boonen B., Alpizar Y.A., Meseguer V.M., Talavera K. (2018). TRP Channels as Sensors of Bacterial Endotoxins. Toxins.

[104] Mazgaeen L., Gurung P. (2020). Recent Advances in Lipopolysaccharide Recognition Systems. Int. J. Mol. Sci..

[105] Han H., Yi F. (2013). New insights into TRP channels. Channels.

[106] Meseguer V., Alpizar Y.A., Luis E., Tajada S., Denlinger B., Fajardo O., Manenschijn J.-A., Fernández-Pena C., Talavera A., Kichko T. (2014). TRPA1 channels mediate acute neurogenic inflammation and pain produced by bacterial endotoxins. Nat. Commun..

[107] Casas J., Meana C., López-López J.R., Balsinde J., Balboa M.A. (2021). Lipin-1-derived diacylglycerol activates intracellular TRPC3 which is critical for inflammatory signaling. Cell. Mol. Life Sci..

[108] Murando F., Peloso A., Cobianchi L. (2019). Experimental Abdominal Sepsis: Sticking to an Awkward but Still Useful Translational Model. Mediat. Inflamm..

[109] Korneev K.V. (2019). Mouse Models of Sepsis and Septic Shock. Mol. Biol..

[110] Dickson K., Lehmann C. (2019). Inflammatory Response to Different Toxins in Experimental Sepsis Models. Int. J. Mol. Sci..

[111] Startek J.B., Talavera K., Voets T., Alpizar Y.A. (2018). Differential interactions of bacterial lipopolysaccharides with lipid membranes: Implications for TRPA1-mediated chemosensation. Sci. Rep..

[112] Diogenes A., Ferraz C., Akopian A., Henry M., Hargreaves K. (2011). LPS Sensitizes TRPV1 via Activation of TLR4 in Trigeminal Sensory Neurons. J. Dent. Res..

[113] Devesa I., Planells-Cases R., Fernández-Ballester G., González-Ros J.M., Ferrer-Montiel A., Fernández-Carvajal A. (2011). Role of the transient receptor potential vanilloid 1 in inflammation and sepsis. J. Inflamm. Res..

[114] Sugiyama Y., Ishida K., Yoshiyama Y., Tanaka S., Kawamata M. (2023). TRPV1 is involved in abdominal hyperalgesia in a mouse model of lipopolysaccharide-induced peritonitis and influences the immune response via peripheral noradrenergic neurons. Life Sci..

[115] Bodkin J.V., Fernandes E.S. (2013). TRPV1 and SP: Key elements for sepsis outcome?. Br. J. Pharmacol..

[116] De Winter B.Y., Bredenoord A.J., Van Nassauw L., De Man J.G., De Schepper H.U., Timmermans J.-P., Pelckmans P.A. (2009). Involvement of afferent neurons in the pathogenesis of endotoxin-induced ileus in mice: Role of CGRP and TRPV1 receptors. Eur. J. Pharmacol..

[117] Fernandes E.S., Liang L., Smillie S.-J., Kaiser F., Purcell R., Rivett D.W., Alam S., Howat S., Collins H., Thompson S.J. (2012). TRPV1 Deletion Enhances Local Inflammation and Accelerates the Onset of Systemic Inflammatory Response Syndrome. J. Immunol..

[118] Scheraga R.G., Abraham S., Niese K.A., Southern B.D., Grove L.M., Hite R.D., McDonald C., Hamilton T.A., Olman M.A. (2016). TRPV4 Mechanosensitive Ion Channel Regulates Lipopolysaccharide-Stimulated Macrophage Phagocytosis. J. Immunol..

[119] Schappe M.S., Szteyn K., Stremska M.E., Mendu S.K., Downs T.K., Seegren P.V., Mahoney M.A., Dixit S., Krupa J.K., Stipes E.J. (2018). Chanzyme TRPM7 Mediates the Ca^2+^ Influx Essential for Lipopolysaccharide-Induced Toll-Like Receptor 4 Endocytosis and Macrophage Activation. Immunity.

[120] Py B.F., Jin M., Desai B.N., Penumaka A., Zhu H., Kober M., Dietrich A., Lipinski M.M., Henry T., Clapham D.E. (2014). Caspase-11 Controls Interleukin-1β Release through Degradation of TRPC1. Cell Rep..

[121] Agnew A., Nulty C., Creagh E.M. (2021). Regulation, Activation and Function of Caspase-11 during Health and Disease. Int. J. Mol. Sci..

[122] Pereira D.M.S., Mendes S.J.F., Alawi K., Thakore P., Aubdool A., Sousa N.C.F., da Silva J.F.R., Castro J.A., Pereira I.C.P., Silva L.C.N. (2018). Transient Receptor Potential Canonical Channels 4 and 5 MediateEscherichia coli-Derived Thioredoxin Effects in Lipopolysaccharide-Injected Mice. Oxidative Med. Cell. Longev..

[123] Drechsler S., Osuchowski M. (2021). Cecal Ligation and Puncture. Methods Mol. Biol..

[124] Herminghaus A., Picker O. (2021). Colon Ascendens Stent Peritonitis (CASP). Methods Mol. Biol..

[125] Schabbauer G. (2012). Polymicrobial sepsis models: CLP versus CASP. Drug Discov. Today: Dis. Model..

[126] Wanner S.P., Garami A., Pakai E., Oliveira D.L., Gavva N.R., Coimbra C.C., Romanovsky A.A. (2012). Aging reverses the role of the transient receptor potential vanilloid-1 channel in systemic inflammation from anti-inflammatory to proinflammatory. Cell Cycle.

[127] Dalsgaard T., Sonkusare S.K., Teuscher C., Poynter M.E., Nelson M.T. (2016). Pharmacological inhibitors of TRPV4 channels reduce cytokine production, restore endothelial function and increase survival in septic mice. Sci. Rep..

[128] Silverman H.A., Tynan A., Hepler T.D., Chang E.H., Gunasekaran M., Li J.H., Huerta T.S., Tsaava T., Chang Q., Addorisio M.E. (2023). Transient Receptor Potential Ankyrin-1-expressing vagus nerve fibers mediate IL-1β induced hypothermia and reflex anti-inflammatory responses. Mol. Med..

[129] Qian X., Numata T., Zhang K., Li C., Hou J., Mori Y., Fang X. (2014). Transient Receptor Potential Melastatin 2 Protects Mice against Polymicrobial Sepsis by Enhancing Bacterial Clearance. Anesthesiology.

[130] Silva R.C.M.C., Correa L.H.T. (2021). Heme Oxygenase 1 in Vertebrates: Friend and Foe. Cell. Biochem. Biophys..

[131] Serafini N., Dahdah A., Barbet G., Demion M., Attout T., Gautier G., Arcos-Fajardo M., Souchet H., Jouvin M.-H., Vrtovsnik F. (2012). The TRPM4 channel controls monocyte and macrophage, but not neutrophil, function for survival in sepsis. J. Immunol..

